# Technological Improvement Rates and Evolution of Energy-Based Therapeutics

**DOI:** 10.3389/fmedt.2021.714140

**Published:** 2021-09-03

**Authors:** Subarna Basnet, Christopher L. Magee

**Affiliations:** ^1^SUTD-MIT International Design Center, Massachusetts Institute of Technology, Cambridge, MA, United States; ^2^Massachusetts Institute of Technology (MIT) Institute for Data, Systems and Society (IDSS), Cambridge, MA, United States

**Keywords:** tissue, stimulation, neuromodulation, energy, invention, patent, performance, innovation

## Abstract

This paper examines the field of energy-based medical therapies based on the analysis of patents. We define the field as the use of external stimuli to achieve biomedical modifications to treat disease and to increase health. Based upon distinct sets of patents, the field is subdivided into sub-domains for each energy category used to achieve the stimulation: electrical, magnetic, microwave, ultrasound, and optical. Previously developed techniques are used to retrieve the relevant patents for each of the stimulation modes and to determine main paths along the trajectory followed by each sub-domain. The patent sets are analyzed to determine key assignees, number of patents, and dates of emergence of the sub-domains. The sub-domains are found to be largely independent as to patent assignees. Electrical and magnetic stimulation patents emerged earliest in the 1970s and microwave most recently around 1990. The annual rate of improvement of all sub-domains (12–85%) is found to be significantly higher than one we find for an aggregate pharmaceutical domain (5%). Overall, the results suggest an increasingly important role for energy-based therapies in the future of medicine.

## Introduction

Electroceuticals is a burgeoning therapeutic field of bioelectrical and bioelectronics medicine, in which electrical energy is utilized to stimulate electrical pathways in the body to modify biological functions or pathological processes in the body (1, 2). Although the field has seen increased interest in the last decade or so, the use of electrical energy stimulation as a therapeutic modality has been around for several decades. Implantable pacemakers and defibrillators, which use electrical energy to stimulate cardiac chambers and nerve centers, have been discussed and used since the 1960s. Deep Brain Stimulation and pelvic floor stimulation using electrical energy were also investigated in the 1960s (3). In recent decades, electro stimulation is finding applications in new areas such as stimulation of peripheral nerves and central nervous system (brain and spinal cord) for treatment and management of a wide variety of diseases (4). Electrostimulation promises to provide complementary or alternative therapies (e.g., for a patient population which does not tolerate surgeries) for pharmacological and invasive surgical therapies and has generated considerable excitement (5–8). The NIH has established a US$248 million fund to map the electrical wiring of the body and advance the development of new therapeutics. Glaxo SmithKline (GSK) and Verily Life Sciences (an Alphabet company) joined forces to establish Galvani Bioelectronics to enable the research, development, and commercialization of bioelectronic medicines (9).

A survey of the literature (10) demonstrates that scientists and engineers have been developing other therapeutic approaches, harnessing alternative forms of energies—magnetic, microwave, optical, and ultrasound—for stimulating tissues and organ systems. The motivation is, generally, for greater specificity, precision, lesser invasiveness and side effects, or greater suitability for a specific tissue and organ system (11, 12). For example, transcranial magnetic stimulation is preferable as it deposits magnetic energy non-invasively, while optical stimulation of cochlea has greater precision compared with electrical stimulation (13). Together with the electrical energy, these five energy-based modalities form a broad portfolio of energy-based therapeutic approaches.

The goal of the research reported here is to make an objective analysis of stimulation therapies and their future potential for healthcare, including what relative impact they might have. In particular, we use patent data to begin to assess some important questions in this technological area. These questions are:

1. How should researchers meaningfully decompose the overall area?
2. How fast is this area (and its sub-areas) improving?
3. What have been the key developmental steps in each of the subareas over the past decade or two?
4. How important might this therapy form be for the future of health care and what other therapies might it affect?

Regarding the first of these questions, there is no generally accepted decomposition, but at least three ways for decomposition can be considered. One is to decompose according to the organ system or disease being treated by the stimulation. The second is to differentiate only between brain stimulation and peripheral nerve stimulation. The third method—found most fruitful with patents in this work—is to decompose based upon the energy form used in the stimulation therapy. Neither decomposing by the organ system nor by brain vs. periphery stimulation was found to be effective frameworks for obtaining separate sets of patents for each technology—a necessary step in our overall research. Using this energy-based framework, we decompose the stimulation therapies into five domains: electrical, magnetic, microwave, optical, and ultrasound. Although microwave and optical energy are both electromagnetic energy-based, we treat them as different. This is because their frequencies are vastly different in the electromagnetic spectrum, and their tissue stimulation mechanisms (described in subsequent paragraphs) are distinct as well, thus leading to a significant difference in treatment modalities.

Our second research question involves the rate of improvement of the overall area and the constituent subareas. Methods for making such assessments from patent sets have been developed by Benson and Magee (14, 15) and the more recent work by Triulzi et al. (16) was utilized in this work and in similar previous applications (17–19). Our third research question will be addressed by the main path methodology (20–22) as applied in Magee et al., Park and Magee, and Feng and Magee (18, 22, 23). In order to address the fourth research question, assessment of improvement rates in pharmaceutical technologies was also pursued *via* patent set analysis so we could compare that strongly established medical therapy path with the energy-based therapies focused upon in this research. Since these methods are applicable to sets of patents, they are not capable of identifying specific future details of therapies. Their value lies in aiding strategic and/or policy decisions such as resource allocations to different areas or personnel and other investments to make in public or private research and development activities.

The following section covers the methodological details; here, we present a brief description of each of these energy-based therapeutic approaches and the distinctions among them.

**Figure 1:**
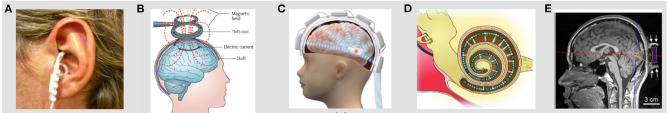
Illustrations of exemplar technologies in energy-based therapeutic domains: **(A)** electrical stimulation of vagus nerve (28); **(B)** transcranial magnetic stimulation of brain (29); **(C)** microwave hyperthermia of brain (30); **(D)** optical stimulation of cochlea with near infrared light (31); **(E)** brain stimulation with focused ultrasound (FUS) (32).

Electrical energy-based therapeutic technologies achieve their therapeutic outcomes by administering electrical current to excitable cells and tissues (1, 24–26). Examples of therapeutic devices are cardiac pacemakers and defibrillators (external and implanted) (24), deep brain stimulators (24, 27), cochlear implants (restoration of hearing), functional electrical stimulators (e.g., spinal cord injury), vagus and sacral nerve stimulators (2, 25, 26). See Figure 1A for an illustrated example. The pacemakers stimulate one or more cardiac chambers by discharging an electrical current for arrhythmia, while external defibrillators stimulate by discharging current across the thorax to control or correct irregular heartbeats. Deep brain stimulation (DBS), an invasive neurosurgical procedure that enables access to inner parts of the brain, involves implanting and securing electrodes in specific structures of the brain stereotactically, while the connecting wires and the pulse generator (a pacemaker-like device) are implanted beneath the skin. The pulse generator is programmed by an external electro-modulator and transmits high-frequency electrical pulses to the electrode to stimulate the target tissue (33). Although its mechanism of action is not well-understood yet, it is believed that it acts by shifting the frequency of oscillatory activity in the brain, for example, in Parkinson's disease, from low to high frequency. The current DBS systems in practice are preprogrammed and operate in open-loop fashion and do not adapt if the symptoms of the patient and underlying physiological parameters shift. To address this shortcoming, new adaptive closed loop DBS systems are being developed, which utilize feedback signals from electrophysiological measurements, neurochemical sensing, or external sensors (such as the use of accelerometers to measure tremors in essential tremors) to shift the stimulation parameters (34–36), enabling the DBS systems to be responsive to the symptoms and needs of the patient.

Transcranial direct current stimulation, a viable non-invasive alternative technology, allows reversible modulation of activity, in particular brain regions, and involves the application of weak electrical current (e.g., 1–2 mA) for a short duration using two (or more) electrodes, anodes, and cathodes on the scalp of the subject, in which one functions as the target electrode and the other as the reference electrode. The target electrode could be either the anode or the cathode, depending on the application (37, 38). The applied current from the electrodes passes through the brain and upregulates or downregulates the cortical areas of the brain as required by the intended therapeutic application. The anodal stimulation depolarizes neurons, increasing the likelihood of occurrence of an action potential, whereas the cathodal stimulation hyperpolarizes the neurons, decreasing the likelihood of occurrence of the action potential.

Vagus nerve stimulation targets the vagus nerve, a mixed parasympathetic nerve, containing ~80% sensory and 20% motor efferent fibers. With extensive branches and sub-branches, the vagus nerve innervates many parts of the human body, ranging from ear, larynx, pharynx, bronchi, lungs, heart, and esophagus, the stomach proximal and descending colon, duodenum, and pancreas (28, 39, 40), thus attracting the attention of many researchers for therapeutic stimulation. In implantable vagus nerve stimulation such as in the treatment of epilepsy and depression, an electrode extending from the pulse generator is wrapped around the left cervical vagus nerve and delivers 20–30 Hz pulse, lasting for 30–90 s (40). In recent years, non-invasive vagus nerve stimulation (VNS) systems have been introduced in which the vagus nerve is stimulated transcutaneously, for example, in auricular regions or on the side of the neck of the subject. The device has two electrodes that are in contact with the skin during the treatment. The weak current travels between the two electrodes, which are in contact with the skin, stimulating the vagus nerve as the current passes through it.

In magnetic energy-based therapeutic technologies, extracorporeal coils (through which a strong electrical pulse is passed) produce a magnetic field pulse, which penetrates the tissue and bone with minimal attenuation and induces eddy currents in the underlying superficial neuronal tissue (which has charged particles), following the induction principle of Faraday (41, 42). The induced current can excite or inhibit electro-neuronal activation by depolarizing or polarizing the cell membranes. Transcranial magnetic stimulation (TMS) is one of the major areas within this domain, in which the different parts of areas in the brain are stimulated non-invasively, using a magnetic field (43). See Figure 1B for an illustrated example. Repetitive transcranial magnetic stimulation (rTMS), a novel approach within TMS with considerable promise, stimulates with repetitive pulses (instead of single) at low and high frequencies. Low-frequency stimulation (<1 Hz) produces long-lasting inhibition of cell-cell communications, known as long-term depression, while the repeated high-frequency stimulation (1–20 Hz or higher) produces improved cell-cell communication by long-term potentiation (44–46). Various coil design configurations— of Figure 8, double angulated coil-forming obtuse angle, Hesed coil, C-core coil, and circular coil—provide a range of options to obtain a combination of desired focus and depth as the application dictates. TMS has shown promise in the treatment of stroke, Parkinson's disease, dystonia, tinnitus, neurogenic pain, epilepsy, amyotrophic lateral sclerosis, depression, anxiety disorders, schizophrenia, addiction and craving, obsessive-compulsive disorder, and memory dysfunction. An emerging non-invasive approach, low-intensity, extremely low-frequency magnetic field stimulation (ELF-MF) is offering another avenue for stimulating the brain. Researchers have studied the influence of ELF-MF on the excitability of the human brain, using a range of frequencies (0–300 Hz, but most below 100) and extremely low intensity (in milli- and micro-Tesla), and the experimental data suggest it could affect pain sensitivity, motor system (e.g., standing balance, postural tremors), cognitive functions (e.g., reaction time, memory, visual discrimination, and flexibility), and could provide non-invasive tools for the treatment of neurologic and neuropsychiatric disorders (47, 48). Another exemplar area not involving the central nervous systems is the non-invasive and passive magnetic stimulation of the pelvic floor or the sacral roots to treat urinary incontinence (stress, urgency, continuous, neurogenic, insensible) (49–52). The pulsed magnetic field produces eddy currents in the excitable tissue and depolarizes the motor nerve to produce an action potential that triggers muscle contractions (53, 54).

Microwave and radiofrequency-based technologies achieve their therapeutic outcomes by heating the target tissue to a cytotoxic level such that the tissue gets ablated (destroyed) or coagulated. In microwave ablation, an alternating electromagnetic field is applied to a tissue to heat it through the mechanism of dielectric heating with the tissue functioning as the dielectric (55, 56). See Figure 1C for an illustrated example. The alternating EM field oscillates the water molecules, in the process of converting a portion of this energy into heat. In radiofrequency ablation, the high-frequency current is passed through the tissue, using ions in the tissue as carriers of charge and completing the electrical circuit. As the current passes through the tissue, the tissue generates heat due to its resistance, using the Joule effect. The close vicinity of the current applicator is directly heated by RF heating, while the larger peripheral area is heated through the conduction of thermal energy from the heating zone. It should be noted the major difference between microwave and RF heating is that microwave heating occurs in a volume around the applicator antenna, while RF heating is limited to areas of high-current density (55–57).

Optical energy-based therapeutic technologies achieve their outcomes by activating biological compounds, cells, and tissues. The activation can occur through dislodging a compound that becomes biochemically active and binding to a downstream effector. An alternative approach is the activation of a light-sensitive protein such as channelrhodopsin, which can then excite the cell to express the opsin (58–60). Depending upon the applications, the optical energy may be supplied by a laser or a near Infra-red light and can use high or low-level intensity. Prior publications have shown possible applications of optical energy-based therapeutics (e.g., transcranial low-level light/laser therapy) to modulate neurological and psychological functions, treat stroke, Parkinson's disease, Alzheimer's disease, chronic neurogenerative conditions, depression, retinal diseases, etc. (61, 62). See Figure 1D for illustrated examples in this subarea.

Ultrasound-based therapeutic technologies achieve their therapeutic outcomes through thermal and mechanical effects produced by ultrasound radiation (63). Thermal effects can produce hyperthermia or ablation of the tissue, depending on the intensity of the radiation utilized. As the ultrasound travels through the tissue, the variation in pressure leads to the shearing of tissue, and friction converts the acoustic energy into heat. Another source of heating is supraharmonic leakage of wave energy into the tissues. Glioblastoma multiforme (GBM), a common and aggressive malignant central nervous system tumor, is treated using ultrasound to produce coagulation, using the thermal effect of ultrasound (63). In pulsed high-intensity focused ultrasound (HIFU), a mechanical effect occurs due to cavitation, which occurs during negative pressure cycles generating gaseous bubbles, and, predominantly, at the tissue interfaces. The mechanical effects are produced in two ways: the stable oscillation of gaseous bubbles and by bursting of these bubbles generating broad band acoustic energy (63, 64). Quadri et al. (63) describe the application of HIFU for the treatment of ischemic stroke, in which HIFU causes microbubble oscillation, leading to a mechanical disruption of the ischemic clot and improved recanalization. The oscillation of these bubbles also contributes to thermal effects. The ultrasound-based therapies have been used and are being investigated for use in neurosurgical and dermatological applications (65–67). Figure 1E illustrates an example of a neurosurgical application. Prominent exemplar applications are brain tumor ablation, treatment of neuropathic pain, movement disorders (such as Parkinson's disease, tremors, and dystonia), immunomodulation, neuromodulation, epilepsy, targeted drug delivery, and adipose tissue removal.

## Materials and Methods

### Patent Sets and Search Methodology

The current research utilizes patents granted by US Patent and Trademark Office (USPTO) to single out six sets of patents as the key data: The first five sets represent the five energy-based therapeutic domains, and the sixth set represents pharmaceutical technologies as a broad category or single domain. These patents sets were constructed using the classification overlap method (COM) and Patsnap patent database (14, 15) to determine the patents in a given domain.

#### Classification Overlap Method and Patent Database

The procedure utilizes US patent classification (UPC) and international patent classification systems (IPC) and typically includes the following steps: (1) identifying a seed patent set using keywords that describe the technological domain of interest; (2) binning the patents in the seed set into IPC (or CPC) and UPC classes; (3) calculating mean-precision-recall (MPR) value for each class, and then ranking classes using these values (this is repeated for the UPC classes); (4) pairing top IPC (or CPC) and UPC classes and retrieving new sets of patents that are listed in both of the top classes from each classification system; (5) reading a sample of patents from these sets to determine the relevancy of these patent sets for the technological domain in question and choosing the best set. The seed sets may also be found using other means such as a list of patents, inventors, or assignees who are known to work in the technological domain. The primary goal of the seed set is to find the relevant IPC (or CPC) classes and UPC classes in the two patent classification systems and use these classes to determine the patents relevant to the domain. Sometimes, when the classes or subclasses do not provide sufficient resolution, keywords may also be used additionally as filters to obtain a more relevant set of patents. To determine the relevancy of a patent set, a sample of 300 patents for each domain is read by two readers independently. Out of the 300 patents, the first 100 patents included those which had received the highest numbers of forward citations in the first 3 years after they were granted, and another 200 patents were randomly selected from the remaining patents in the patent set for the domain in consideration. Publications by Benson and Magee (14, 15) provide in-depth discussion and further details of the method used here.

The current study utilized the commercial patent database PatSnap, which provides patents granted by the USPTO from 1970 onwards in an electronic format. This study considered all utility patents granted from January 1, 1970 to February 15, 2017 to retrieve patents relevant to each domain. The seed patent set for each technological domain was decomposed into UPC and IPC classes. The top five UPC and IPC classes (or subclasses) with the highest MPR values were utilized to operationalize the overlap (common patents) between the two classification systems. Table 1 shows the final set of IPC and UPC classes used for classification overlap to retrieve patents relevant to the five energy-based therapeutic domains along with the number of patents retrieved and the relevancy score of the patent set for the respective domains. We note that it was sufficient to include only one class or subclass from the UPC and the IPC for operationalizing the classification overlap method (COM) for electrical and ultrasound therapeutic domains. In contrast, the other three therapeutic domains—optical, magnetic, and microwave—required a combination of two or more UPC and IPC subclasses to obtain a complete set of patents, with the magnetic and microwave therapeutic domains requiring the most as shown in Table 1.

**Table 1:** Energy-based therapeutic technological domains with the respective UPC and IPC classes used for obtaining patents between 1970 and 2015, using the COM method.

**Therapeutic Domains**	**COM Classes (UPC AND IPC)**	**Number of patents**	**Relevancy%**
Electrical	UPC:(607) and IPC:(A61N1/05)	4,613	90
Magnetic	UPC:(600/9 or 600/12 or 600/13 or 600/14) and IPC:(A61N2/00 OR A61N2/02 OR A61N2/0)	658	98
Microwave/RF	UPC:(606/33 or 607/101 or 607/102) and IPC:(A61B18/18 or A61N5/02 or A61N5/04)	1,096	90
Optical	UPC:(607/88 or 607/89) and IPC:(A61N5/06 or A61N5/067)	1,140	92
Ultrasound	UPC:(601/2) and IPC:(A61N7/00)	390	93

*The number of patents and relevancy of each patent set is also presented*.

#### Patent Sets for Five Energy-Based Therapeutics and Their Characteristics

The electrical therapeutic domain has more than 4,600 patents, the largest among the five domains. The microwave/RF and optical have about 1,200 patents, 1/4 of the number of patents in the electrical therapeutic domain. The magnetic and ultrasound therapeutic domains have about 700 and 400 patents, respectively, one-seventh and one-twelfth the number of patents in the largest patent set. Even the smallest set for ultrasound is large enough to allow a reliable study of the domain (14, 15). The relevancy score for all the five domains is 90% or greater, which is substantially higher than the cutoff value of 75% for a patent set to be suitable for analysis of a technological domain (14, 15). The patent sets for these five domains are provided in Supplementary Information for reference.

#### Common Patents Between Pairs of Energy-Based Therapeutic Domains

**Figure 2:**
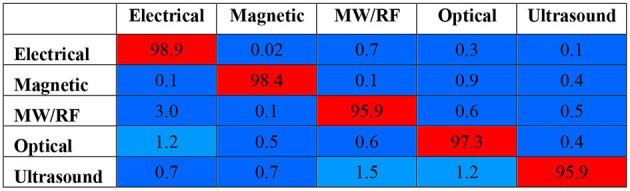
Heat map of percentage of common patents between domains. A diagonal cell shows the percentage of patents in a focal domain (e.g., 97.3% for optical stimulation in the fourth row) that are not common with any of the other domains. The off-diagonal cell indicates the percentage of patents in a focal domain common with another domain (e.g., 0.6% for optical stimulation with Microwave/RF stimulation).

The common patents are quantified as the percentage of patents common between two patent sets, with each patent set representing a technology domain. The extent of commonality between two different patent sets representing different technological domains indicates the degree of independence between the two domains and indicates whether we can treat these separately. In those cases where commonality is larger, it also implies that patents might have applications in different functional areas. The heat map in Figure 2 shows the ratio of patents in the focal domains (row headings) that are common with another domain (shown in the column headings) to the number of patents in the focal domain itself. For example, the optical therapeutic domain, the focal domain in row 4, has only 0.6% (an off-diagonal cell in row 4 and column 3) of its patents that are common with the microwave/RF domain. Each of the other off-diagonal cells can be interpreted similarly. The diagonal cells for the focal domain (e.g., 97.3% for optical stimulation in the fourth row) show the ratio of patents that are *not* common to any of the other four domains. Note that the percentage of patents that are common between different therapeutic domains is very low, with the highest value being only 1.5% for microwave/RF therapeutic (the focal domain) with the ultrasound therapeutic domain, suggesting a high degree of independence between the five therapeutic domains. This degree of independence is also reflected by the high values in the diagonal cells or all the focal domains.

#### Patent Sets for Pharmaceutical Domains

We utilize three pharmaceutical therapeutic domains—Alzheimer's and Parkinson's diseases, cardiovascular diseases, and respiratory diseases—which are selected based on an anatomical therapeutic chemical classification system (ATC) as a context and as a baseline to compare their annual improvement rates with those of energy-based therapeutic domains. It should be noted that these domains are not the primary subject of this study. We utilized the patent sets developed by Guo, Park, and Magee for these pharmaceutical domains (68), using the Patsnap database. Table 2 summarizes the UPC and IPC classes used for implementing the COM procedure, and the resulting number of utility patents along with their relevancy scores. It should be noted that the authors used mostly three-digit classes, which are at a higher level in both the classification systems. This is appropriate as they are considering *all* patents relevant to a particular anatomical system (e.g., cardiovascular system). Consequently, the size of the patent sets was much higher than the ones we had for energy-based therapeutics (compare Tables 1, 2). The relevancy score, determined using the same procedure as the one described above for energy-based therapeutics, is in the mid-80s, higher than the cut-off level of 75%.

**Table 2:** Pharmaceutical therapeutic technological domains with their respective UPC and IPC classes used for obtaining patents between 1976 and 2015, using the COM procedure.

**Pharmaceutical therapeutic domains**	**COM classes (UPC and IPC)**	**Number of patents**	**Relevancy %**
Alzheimer's & Parkinson's diseases	UPC:(424 or 514) and IPC:(A61P25/28 or A61P25/16)	6,331	83
Cardiovascular disease	UPC:(424 or 514) and IPC:(A61P9)	14,361	84
Respiratory disease	UPC:(514) and IPC:(A61P11)	6,396	92

*The number of patents and relevancy of each patent set is also presented*.

**Figure 3:**
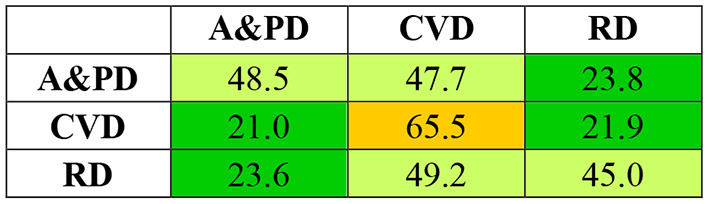
Heat map of percentage of common patents between pharmaceutical domains. Alzheimer's and Parkinson's disease (A and PD), cardiovascular disease (CVD), and respiratory disease (RD). See Figure 2 for instructions to interpret the numbers.

To understand the independence of these pharmaceutical domains, we considered the overlap of patents between these domains. The heat map in Figure 3 shows the percentage of patents in the focal domains (row headings) that are common with another domain (shown in the column headings), calculated with respect to the number of patents in the focal domain itself. The numbers in the cells in the embedded table should be interpreted as the cells in the Figure 2 (please see the Figure 2 for further instructions for interpreting the numbers). In contrast to the energy-based domains, each pharmaceutical domain shows much more overlap with other domains; only 50–65% of the patents in each domain are *not* common with other domains. Both Alzheimer's and Parkinson's diseases (A and PD) and respiratory diseases (RD) domains have close to 50% of the patients common with cardiovascular diseases (CVD). However, between A and PD and RD domains, common patents are only in the low 20%. It is not surprising to see such overlap, as it is a common practice in the pharmaceutical industry for a given treatment to have multiple disease applications (69–71). Because of such high overlap, we considered the pharmaceutical patents as an aggregate set and calculated the annual improvement rate for the pharmaceutical domain based on the aggregate set.

### Centrality and Estimating Annual Improvement Rates

The estimation of annual improvement rate for a patent set for a technological domain is based on the average normalized centrality of patents in the set (16). The centrality of a patent is analogous to the betweenness centrality (BC) in a network, which, in our case, is the patent citation network. The BC measures the number of times a patent (a node in the citation network) lies on the shortest path between two other patents (nodes) in the citation network and provides a metric to estimate the influence a patent (node) has in the flow of technological knowledge (information). The calculation of BC in our study has its origins with Hummon and Doreian (20) and their introduction of search path node pairs (SPNP) as a metric to compute the BC of a focal paper in a scientific paper citation network. The SPNP for a focal patent (say, patent B) in the patent citation network counts the number of pathways originating from one patent (say, patent A) to another one (patent C) *and* passes through the focal patent (patent B). The higher the number of pathways passing through the focal patent, the higher the centrality of the patent, indicating the importance of the patent. Interpreting each patent as introducing some original technological knowledge, the centrality provides an indication of the significance of the original knowledge introduced by the focal patent for the downstream patents. As an extension of the method, Triulzi et al. (16) normalized the SPNP to account for the variations introduced by changes in citation practices between domains and over time. Such variations make the raw centrality estimates of patents difficult to compare. To overcome these difficulties, Triulzi et al. compare the estimated centrality of a patent in the patent citation network with the estimated centrality of the same patent in a randomized model of the patent citation network (16). They utilize all utility patents from 1976 to 2015 and their citations in the United States Patent System to develop the patent citation network to compute the centrality of the patents. Triulzi et al. further found that the mean normalized centrality of patents in a patent set representing a specific technological domain is a reliable predictor of its annual rate of improvement (*k*). They have concluded this finding with a Monte Carlo cross-validation exercise between empirically observed k for the 30 diverse technological domains (16) and their corresponding mean normalized centrality of the patent sets for the same 30 technological domains. Their regression model is shown below:


$$\begin{array}{l} k_{i}=Exp\left(-5.01885*C_{i}+\;\frac{\sigma_{i}^{2}}{2}\right) \end{array}$$

where *k*_*i*_ represents the annual rate of improvement for domain i; *C*_*i*_, the mean normalized centrality of the patent set for domain *I*; and σ_*i*_, *the standard deviation*. We use their regression model to estimate the annual improvement rates (*k*) for the five energy-based therapeutic domains and the aggregate pharmaceutical domain.

### Main Path Methodology

Main path methodology furnishes the means to determine significant patents in a patent set for a technological domain, where the significant patents collectively act as pathways through which technological knowledge advances in the domain. The most important reason for the use of the technique is to identify a *readable* number of significant patents in the domain and to enable the identification of technology clusters within the domain. Hummon and Doreian (20) first used the method to understand the evolution of scientific fields through the study of citations by scientific publications. Later, many researchers (21, 72, 73) utilized and adapted the method to understand the advancement of many technological domains. More recently, Park and Magee (22) have optimized the method to generate simpler main paths, while capturing a greater number of significant patents. Labeled as genetic-backward-forward path (GBFP) analysis, the optimized method includes four steps: (1) collecting a patent set for a technological domain; (2) developing a citation network among patents in the patent set; (3) measuring the knowledge persistence of each patent in the domain patent set and identifying high and low persistence patents; (4) and constructing main paths among the high-persistence patents searching backward and forward from the high-persistent patents. Steps 3 and 4 are discussed in greater detail next.

Step 3 includes three sub-steps: (3.a) structuring the citation network from Step 2 into layers after defining start and end points, (3.b) calculating knowledge persistence of each patent in the domain patent set by considering backward and forward citations of each patent, (3.c) and identifying the patents with global (GP) and local (LP) persistence equal to or >0.3 and 0.8, respectively, as high-persistence patents. The GP of a patent estimates the importance of a patent in the entire network, whereas the LP estimates the importance of the patent in each layer. The local persistence (LP) plays a significant role in identifying and retaining important patents, which are recent but have not had a chance to enable the evolution of their lineage. In Step 4, the main paths are traced backward and forward, originating from the high-persistent patents. Often, it is necessary to connect main pathways traversing through high-persistent patents with low-persistent patents to make the main pathways continuous. As a final note, it should be noted that, in Step 2, the citation network is created only among patents in the domain patent set, and any citations outside the patent set are ignored. In contrast, the citation network constructed in the computation of centrality of patents (and annual improvement rates) includes all the utility patents in the USPTO system. The readers are referred to prior publications for methodological details as well as for applications of the method to other domains (18, 22, 23).

## Results

### Patenting Activity in the Five Energy-Based Therapeutic Domains

**Figure 4:**
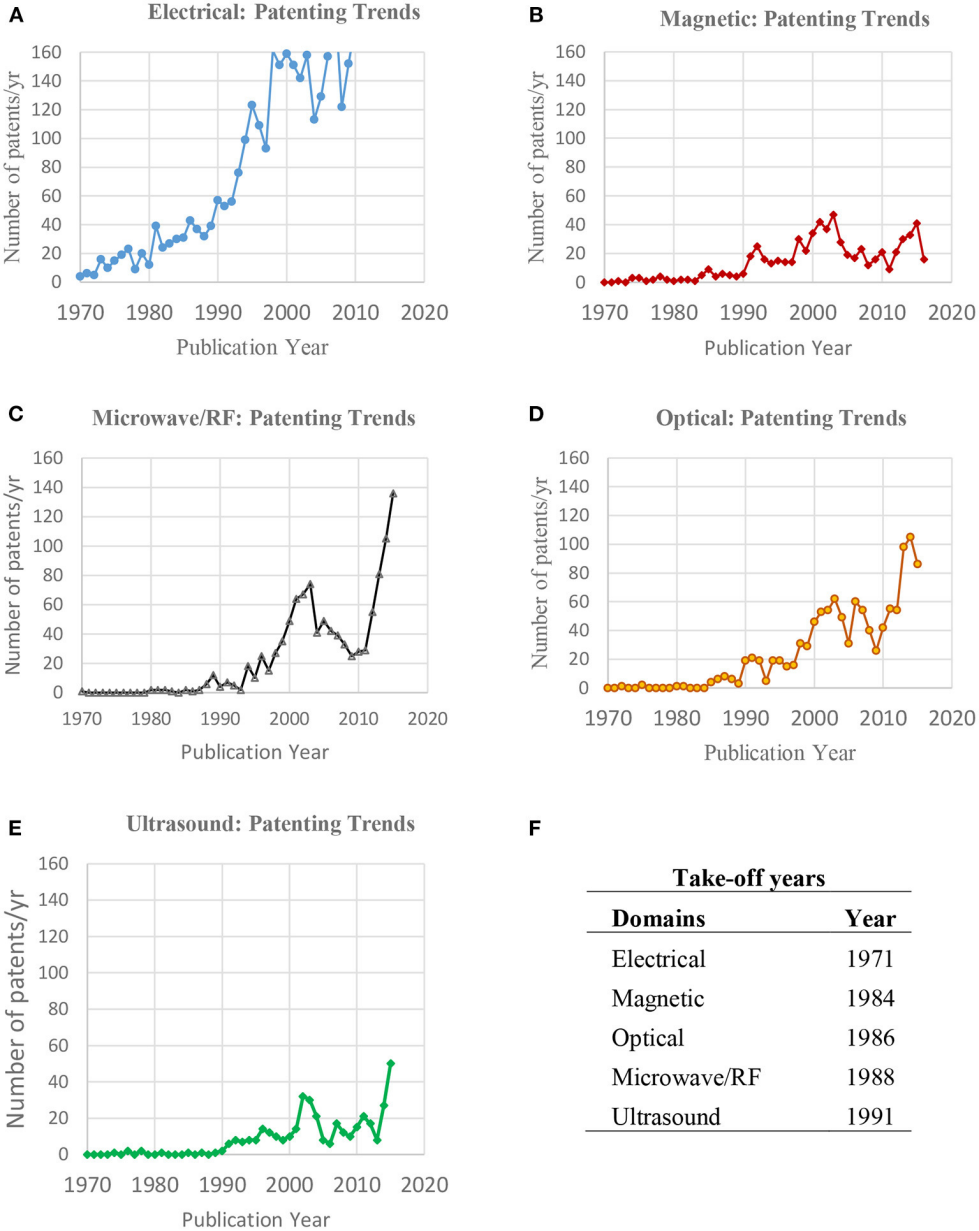
**(A–F)** Patenting trends in the five energy-based therapeutic domains—annual number of patents in **(A)** electrical, **(B)** magnetic, **(C)** microwave/radiofrequency (MW/RF), **(D)** optical, and **(E)** ultrasound. **(F)** Shows the years for each of the five domains when at least five patents per year were granted in the domain for the first time.

Patent activity in a technological domain indicates the level of interest in the technological domain and helps to identify when patenting activity accelerates. Figure 4A through Figure 4E show the number of patents granted annually for the five therapeutic domains from 1970 until 2015. Figure 4F summarizes take-off years for the five domains, where we selected takeoff year as that year when the number of patents granted annually was 5 or more (We also found that the order in which the domains took off did not change if we selected 3 or 4 patents per year as the threshold). The electrical domain was the first to become active from the early 1970s, while the magnetic therapeutic domain did so only a decade and a half later, followed quickly by optical and microwave/RF, and then by ultrasound as the last one 2 decades after the electrical therapeutic domain. The electrical therapeutic domain shows the most activity overall, with cardiac pacing technologies starting early in the 1970s contributing significantly to the growth of this technological domain. The microwave/RF and optical therapeutic domains are second in activity, followed by magnetic and ultrasound therapeutic domains. It should be noted that all five domains show slow growth from 2000 to just after 2010, after which they show increased activity again.

Tables 3–7 list the five most active patent assignees in each of the five energy-based therapeutic domains. In the electrical therapeutic domain, Medtronic is clearly the dominant leader, with more than 800 patents (close to 17% of the patents in the domain), followed by cardiac pacemakers with over 400 patents (just over 8%). Pacesetters, Inc and Boston Scientific Neuromodulation Corp. come as distant third with around 200 patents. In the magnetic therapeutic domain, Life Resonances, Inc. and Neuronetics, Inc. are leaders and own 16 patents (just over 2%) of the patents in the domains. Electro-biology and Nu-Magnetics both own 10 patents (around 1.5%). Unlike in the electrical therapeutic domain, the leading assignees in the magnetic therapeutic domain are not as dominant. In the Microwave/RF domain, Covidian is the clear leader with over 100 patents (close to 10% of the patents in the domain). Boston Scientific Scimed Inc. comes second with less than half the number of patents (<4% of the patents in the domain), followed by Vivant Medical, Rita Medical Image, and Thermage. In the optical therapeutic domain, Palomar Technologies and Lockheed Corporation^1^ lead the domain with 25 and 22 patents, respectively (just over 2% of the domain), followed closely by the General Hospital Corporation (doing business as Massachusetts General Hospital) with 17 patents (just about 1.5% of patents). Note this domain shows similar activity trends for the assignees as the magnetic therapeutic domain. Lastly, in the ultrasound therapeutic domain, Exogen is the leader with 25 patents (over 6% of the patents in the domain), followed by Siemens Aktiengesellschaft with 15 patents (over 3.5%). The University of California, Kona Medical and Guided Therapy Systems, each owns close to 10 patents. Overall, the patent ownership is concentrated in the first two assignees in the electrical and microwave/RF domains, while it is much more distributed in the other three therapeutic domains.

**Table 3:** Top 5 current assignees in the electrical energy–based therapeutic domain.

	**Top five current assignees**	**# Patents**
1	Medtronic, Inc.	819
2	Cardiac Pacemakers, Inc.	408
3	Pacesetter, Inc.	206
4	Boston Scientific Neuromodulation Corp.	197
5	Advanced Neuromodulation Systems, Inc.	94

**Table 4:** Top current assignees in the magnetic energy-based therapeutic domain.

	**Top five current assignees**	**# Patents**
1	Life Resonances, Inc.	16
2	Neuronetics, Inc.	16
3	Electro-Biology, Inc	10
4	Nu-Magnetics, Inc.	10
5	Amei Technologies Inc.	6

**Table 5:** Top current assignees in the microwave/radiofrequency energy-based therapeutic domain.

	**Top five current assignees**	**# Patents**
1	Covidien Lp	110
2	Boston Scientific Scimed, Inc.	43
3	Vivant Medical Inc.	36
4	Rita Medical Systems, Inc.	33
5	Thermage, Inc.	31

**Table 6:** Top current assignees in the optical energy-based therapeutic domain.

	**Top Current Assignees**	**# Patents**
1	Palomar Medical Technologies	25
2	Lockheed Corporation	22
3	The General Hospital Corporation	17
4	Ceramoptec Industries	12
5	Koninkline Philips N. V.	12

**Table 7:** Top current assignees in the ultrasound energy-based therapeutic domain.

	**Top five current assignees**	**# Patents**
1	Exogen, Inc.	22
2	Siemens Aktiengesellschaft	15
3	Kona Medical, Inc.	10
4	The Regents of the University of California	10
5	Guided Therapy Systems, LLC	9

### Central Patents

Tables 8–12 list the five patents with the highest normalized centrality values within the patent set for each therapeutic domain. The tables also present the corresponding assignees for each patent. The centrality value of a patent in the entire USPTO patent citation network indicates the frequency of a given patent is in different pathways between any two patents in the entire citation network of utility patents (using the techniques described in the Material and Methods section), and, as such, central patents represent the most important patents for the flow and advancement of overall technological knowledge. In the electrical therapeutic domain, the four patents (listed as 1, 3, 4, and 5 in Table 8) are inventions for stimulating the nervous system, and while the other one (listed as 2) is for stimulating the gastric wall to treat gastrointestinal disorders. It is interesting to note that the assignees for these central patents are *not* among the five most active assignees in Table 3. However, Medtronic, the most active assignee, does own the 7–9th most central patents in the electrical therapeutic domain (not listed in Table 8 to conserve space).

**Table 8:** Top five central patents for the electrical energy-based therapeutic technological domain with their assignees and centrality values.

	**Publication number**	**Title**	**Assignee**	**Centrality**
1	US6516227	Rechargeable spinal cord stimulator system	Advanced Bionics Corporation	0.998
2	US6535764	Gastric treatment and diagnosis device and method	Intrapace, Inc.	0.998
3	US6587719	Treatment of obesity by bilateral vagus nerve stimulation	Cyberonics, Inc.	0.997
4	US6185452	Battery-powered patient implantable device	Gord John C. | Dell Robert Dan | Schulman Joseph H.	0.997
5	US6885888	Electrical stimulation of the sympathetic nerve chain	The Cleveland Clinic Foundation	0.997

**Table 9:** Top five central patents for the magnetic energy-based therapeutic technological domain with their assignees and centrality values.

	**Publication number**	**Title**	**Assignee**	**Centrality**
1	US6402678	Means and method for the treatment of migraine headaches	Neuralieve, Inc.	0.981
2	US6198958	Method and apparatus for monitoring a magnetic resonance image during transcranial magnetic stimulation	Beth Israel Deaconess Medical Center, Inc.	0.973
3	US6425852	Apparatus and method for transcranial magnetic brain stimulation, including the treatment of depression and the localization and characterization of speech arrest	Emory University	0.961
4	US7189198	Magnetically guidable carriers and methods for the targeted magnetic delivery of substances in the body	Stereotaxis, Inc.	0.955
5	US6572528	Magnetic field stimulation techniques	Mclean Hospital Corporation	0.952

**Table 10:** Top five central patents for the microwave and radiofrequency energy-based therapeutic technological domain with their assignees and centrality values.

	**Publication number**	**Title**	**Assignee**	**Centrality**
1	US6849073	Apparatus and method for creating, maintaining, and controlling a virtual electrode used for the ablation of tissue	Medtronic, Inc.	1.000
2	US6506189	Cool-tip electrode thermosurgery system	Sherwood Services Ag	0.999
3	US6514250	Suction stabilized epicardial ablation devices	Medtronic, Inc.	0.999
4	US6413255	Apparatus and method for treatment of tissue	Thermage, Inc.	0.999
5	US6517536	Transmural ablation device and method	Atricure, Inc.	0.999

**Table 11:** Top five central patents for the optical energy-based therapeutic technological domain with their assignees and centrality values.

	**Publication number**	**Title**	**Assignee**	**Centrality**
1	US6508813	System for electromagnetic radiation (different types of energy) dermatology and head for use therewith	Palomar Medical Technologies, Inc.	0.987
2	US6443978	Photomatrix device	The University of Arkansas	0.981
3	US6997923	Method and apparatus for EMR treatment	Palomar Medical Technologies, Inc. | The General Hospital Corporation	0.974
4	US6517532	Light energy delivery head	Palomar Medical Technologies, Inc.	0.969
5	US6290713	Flexible illuminators for phototherapy	Russell Thomas A.	0.968

**Table 12:** Top five central patents for the ultrasound energy-based therapeutic technological domain with their assignees and centrality values.

	**Publication number**	**Title**	**Assignee**	**Centrality**
1	US6012457	Device and method for forming a circumferential conduction block in a pulmonary vein	The Regents of the University of California	0.998
2	US6692450	Focused ultrasound ablation devices having selectively actuatable ultrasound emitting elements …	Medtronic Xomed, Inc.	0.997
3	US5558092	Methods and apparatus for performing diagnostic and therapeutic ultrasound simultaneously	Imarx Pharmaceutical Corp.	0.996
4	US6740040	Ultrasound energy driven intraventricular catheter to treat ischemia	Advanced Cardiovascular Systems, Inc.	0.996
5	US6164283	Device and method for forming a circumferential conduction block in a pulmonary vein	The Regents of the University of California	0.992

In the magnetic therapeutic domain, four of the five most central patents (listed as 1, 2, 3, and 5 in Table 9) focus on inventions for transcranial stimulation of the brain, while the remaining patent (listed as 4) is for an invention to facilitate targeted delivery of drug using magnetic particles. Another four patents among the next five most central patents (not listed in the table) are also focused on the transcranial brain stimulation, demonstrating the level of interest and significance of this application to this treatment method.

In the microwave/RF therapeutic domain, all five patents utilize RF energy for ablation of tissue; out of which, the 4th is for ablating skin tissue. Medtronic Inc., the most active assignee, also owns two (1st and 3rd) of the five most central patents in the domain, plus the 10th patent (not listed in Table 5).

In the optical therapeutic domain (Table 11), four of the five most central patents (1, 2, 4, and 5) are technologies for efficient delivery of laser or optical radiation to target tissue and include management of backscattering of light and cooling of the device or the tissue exposed to the light. Although not among the top five most central patents, three patents among the next five most central patents (US6663659, US6494900, and US6471716) focus on stimulation of tissues. Palomar Medical Technologies, the most active assignee in this domain, is the assignee for three of the five most central patents in this domain.

In the ultrasound therapeutic domain, ablating or making lesions inside tissue or through the organ wall is the focus of the five most central patents. The Univerisity of California system, the fourth most active assignee, owns two of the five most central patents (listed as 1 and 5 in Table 12). Therus Corporation and Transurgical Inc. each owns two central patents, which are among the next five most central patents. Both of these assignees are *not* among the five most active assignees.

### Performance Improvement

**Figure 5:**
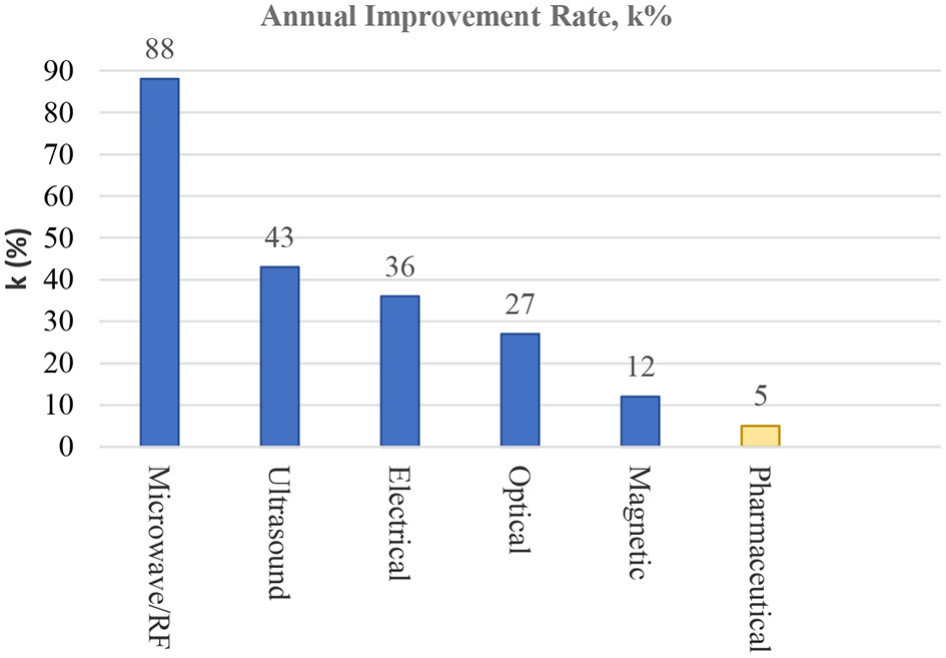
Comparison of annual improvement rates (*k* in %) of the five energy-based therapeutic domains. The *k* values are presented in descending order, and the aggregate *k* value for the pharmaceuticals is presented for reference.

Figure 5 compares the annual performance improvement rates (*k*) of energy-based therapeutic domains (blue bars) and the aggregate pharmaceutical therapeutic domain (orange bar) in descending order. The *k* values are computed using the regression model (described in the Data and Methods section) from the mean normalized centrality of the patent set for a given technological domain. The k value for the aggregate pharmaceutical domain is presented to provide a baseline and a context for comparison to allow some assessment of the growth potential of energy-based therapeutic modalities. The k values for the aggregate pharmaceutical domain are 5% (There is little difference between individual k values for the respiratory diseases, cardiovascular diseases, and Alzheimer's and Parkinson's diseases, and, thus, we used only the k value for the aggregate pharmaceutical set). Earlier analysis of the uncertainty in the k estimates (16, 18) reports that ± 50% uncertainty is reasonable quantification of k ± σ, which indicates the rate of the pharmaceutical domain might be 7.5% on the higher side. Even with this possibility, when we compared this value with the empirically determined k values of 30 diverse domains (74), which range from 6 to 68%, k values for the pharmaceutical domain was low and was similar to the k values of slowly improving domains, such as milling machines and permanent magnetic materials.

Figure 5 shows that k values for the energy-based therapeutic domains range from 13% for the magnetic therapeutic domain to 88% for the microwave/RF domain. Except for the magnetic therapeutic domain, the four domains have medium-to-high improvement rates in comparison to the k values of 30 empirically studied domains (74). In comparison to the pharmaceutical domains, all five energy-based domains are relatively high, with k values 2–16 times higher. It should be noted that the estimated k value of microwave/RF is very high when compared with k values of other energy-based domains. It is in the range of control and software technologies, and, at least, the most central patent in this domain (see Table 11) is consistent with high k value of these technologies. In addition, recent work by Singh et al. (75) indicates a large number of domains with k values beyond 90% per year.

### Main Path Results

The main path analysis determines significant patents in a patent set for a technological domain, which collectively act as pathways through which technological knowledge advances in the domain, and, secondly, the analysis identifies the technology clusters (using techniques described in the Material and methods section). The results on significant clusters and important contributing patents for the five energy-based therapeutic domains are presented below.

Main paths for each of the five domains of interest are presented next. The nodes represent the patents in the domain, and the edges represent the citations, proxy for the flow of knowledge. The larger nodes in yellow color are high-persistent patents, while the smaller gray nodes are low-persistent patents, which are used to connect the high-persistent patents to construct the main paths. The time axis is shown below the main path, and the position of the patent along the time axis indicates the year in which it was granted. The nodes are labeled in ascending temporal order from left to right, with the oldest patent labeled as 1, followed by newer patents toward the right. This labeling scheme is used to show the sequence in which the patents in the main paths were granted. The table below the graphics lists the node labels and patent numbers in the main paths.

#### Main Paths for the Electrical Therapeutic Domain

**Figure 6:**
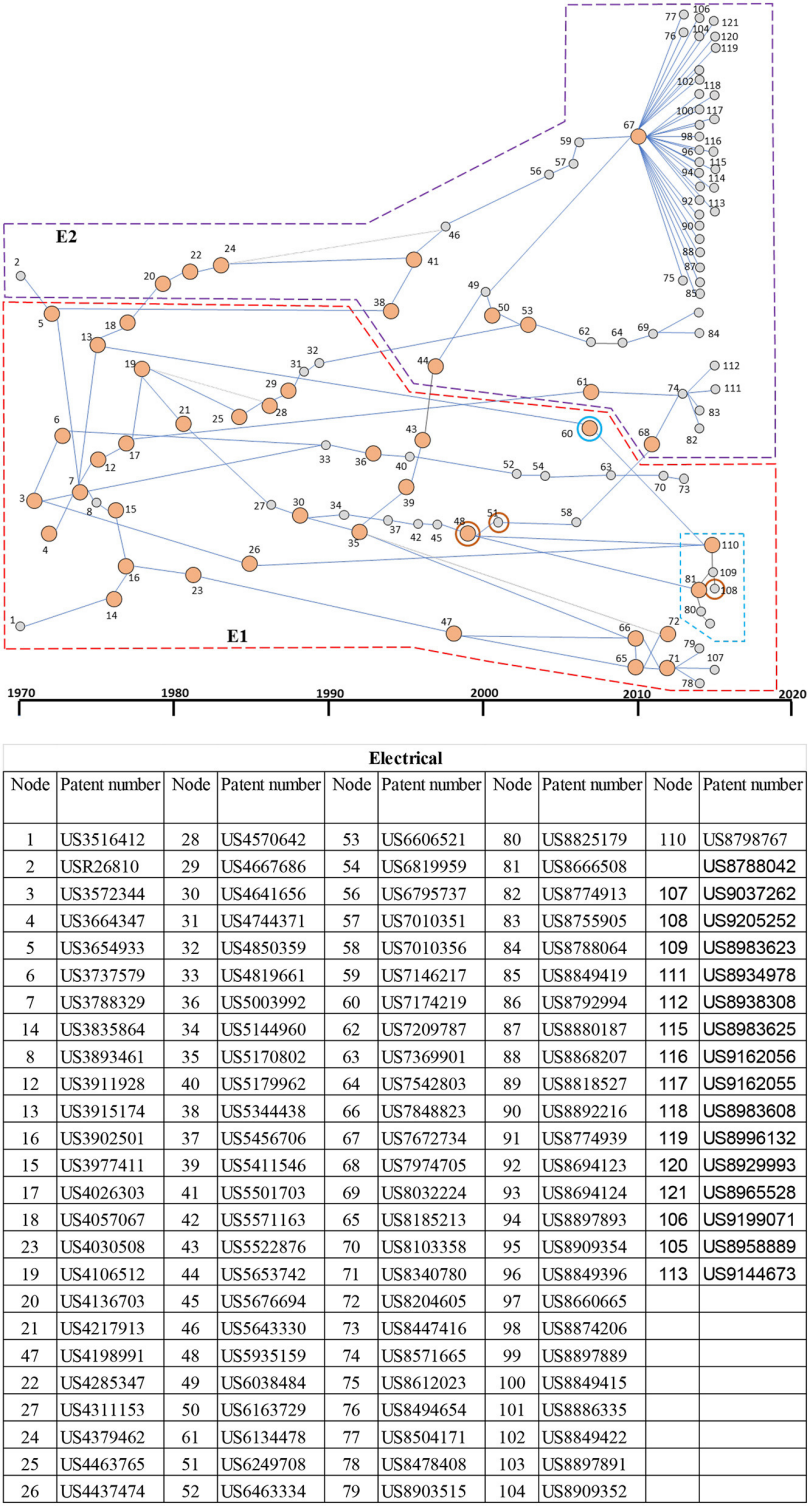
Main paths for the electrical therapeutic domain. E1: A patent cluster for cardiac stimulation for pacing and defibrillation (red box); E2: A patent cluster for neurostimulation (purple box). The blue box shows a subcluster for MRI-compatible leads and electrodes. The patents inside the E1, which are highlighted with red concentric circles, are neuromodulation patents. The table presents details of each patent in the main paths. High-persistence patents are shown as bigger yellow circles, whereas the low-persistence patents are shown as smaller gray circles.

The main paths for the electrical domain show two dominant clusters (E1 and E2 in Figure 6). The first and largest cluster E1, shown as a dashed red box in the figure, is primarily related to cardiac stimulation, and the inventions focus on cardiac pacing and defibrillation. The cluster started from the 1970s (or earlier) and continues to this decade. The key patents in the E1 cluster are US3788329 (node 7) related to the body implantable lead, US3902501 (node 16) related to the endocardial electrode, and US5170802 (node 35) related to the implantable electrode within a blood vessel (all three patents from Medtronic); US3835864 (node 14) related to intracardiac stimulator from Intermedics, Inc; US4198991 (node 47) related to cardiac pacesetter lead from Pacesetter, Inc.; and US8340780 (node 71) related to leadless cardiac pacing from Boston Scientific Scimed, Inc. The E1 cluster set the foundation for the emergence of neuromodulation (using electrical energy), the second cluster E2. The E2 cluster evolved along two main paths, with the first one starting in the1980s (see nodes 22 and 24). Patent US4285347 (node 20) from Cordis Corporation on directional neural lead for spinal stimulation was the first one to make the linkage in the 1980s. The work in neuromodulation was further advanced by US4379462 patent (node 24) on multielectrode catheter assembly for spinal stimulation from Advanced Neuromodulation Systems, Inc. In the 1990s, two patents: US5344438 (node 38 in E2) from Medtronic on the cuff electrode for nerve stimulation and US565374 (node 44 in E2) from Cochlear Limited on bioabsorbable polymers on cochlear implants, with the latter on a different main path interacted with E2 cluster. After 2000s, two patents—US6606521 (node 53 in E2) on the implantable lead for brain stimulation and US7974705 (node 68 in E2) on multiplexed multi-electrodes for neuromodulation—continued to link the E1 and E2 clusters, demonstrating ongoing spillover effect from the E1 cluster. The additional key patent in the E3 cluster, which is US7672734 (node 67) from Boston Scientific Neuromodulation, Inc. on non-linear electrode array, is another key patent, which has given rise to many incremental inventions. It is noted that three patents (nodes 48, 51, and 108) inside cluster E1, marked with bold red circles, are also related to neuromodulation. Furthermore, the patents related to implantable leads and electrodes with MRI compatibility are marked as a sub-cluster, shown as a blue box in Figure 6.

#### Main Paths for the Magnetic Therapeutic Domain

**Figure 7:**
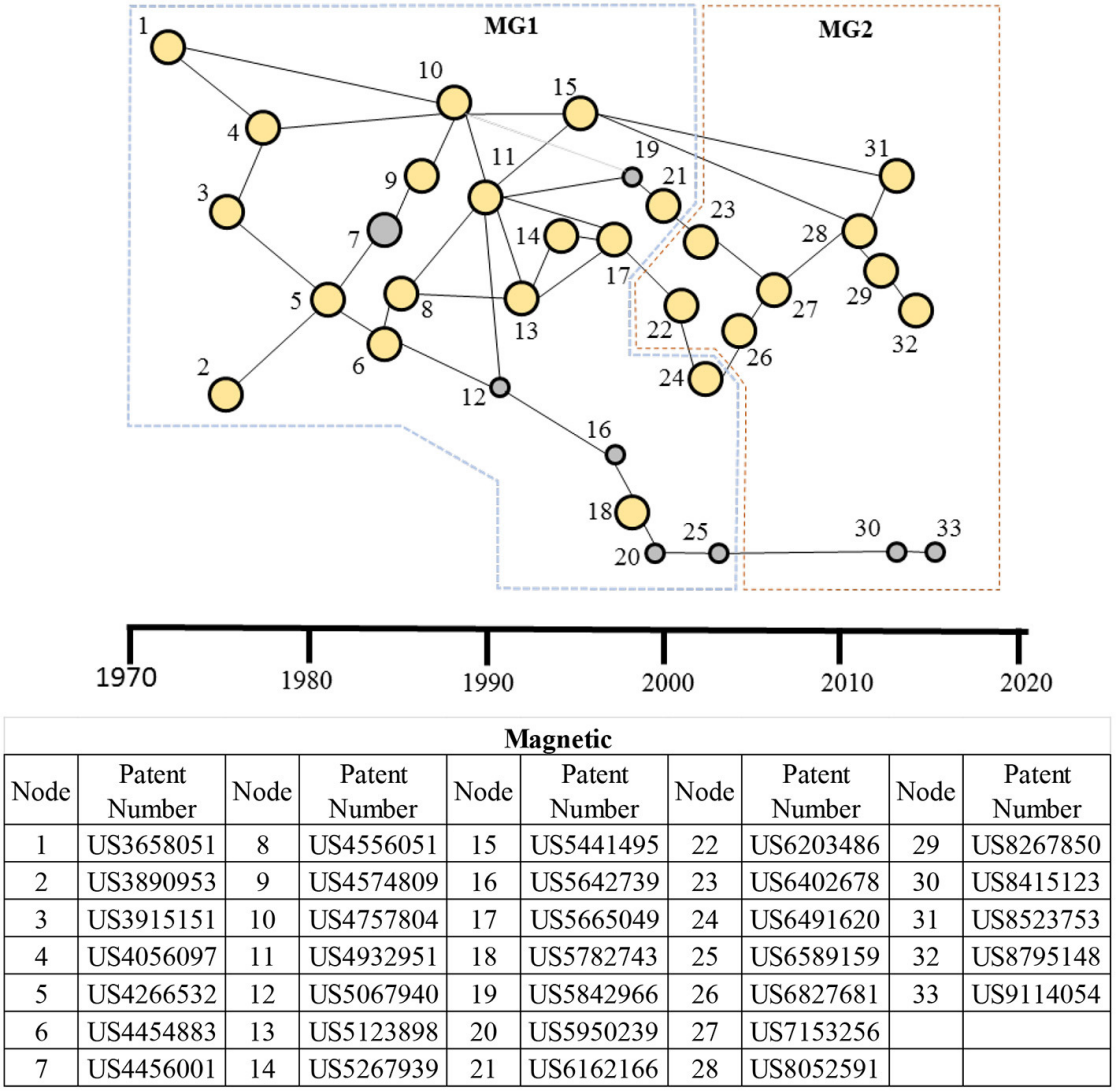
Main paths for the magnetic therapeutic domain. MG1: A patent cluster for stimulation of tissue/bone growth; and MG2: a patent cluster for transcranial brain stimulation. The table presents details of each patent in the main paths. High-persistence patents are shown as bigger yellow circles, whereas the low-persistence patents are shown as smaller gray circles.

The main paths for the magnetic domain show two dominant clusters (see Figure 7). The first cluster of patents, MG1 in the figure, is primarily related to stimulation of tissue/bone growth. The cluster began in the early 1970s and grew until the early 2000s. The patents labeled 11 and 13 (US4932951 and US5123898, respectively, and both issued to Life Resonances, Inc.) are key patents in the MG1 cluster and are related to inventions with a magnetic field generator with a field detector that enables maintaining a fluctuating magnetic field with a preselected ratio of frequency to average flux density at the target. The MG1 cluster set the foundation for the second cluster, MG2 (see Figure 7) centered primarily around transcranial brain stimulation to emerge in the early 2000s. US6203486 (node 22 in MG2) on earth magnetic field augmenters and US6402678 (node 23) on EM therapy for treating migraines linked MG2 to MG1 clusters in 2000s. The node 28 (US8052591 also from Life Resonances) in the MG2 cluster is a key patent related to stereotactic (accurate positioning of probes using 3D diagrams) transcranial magnetic stimulation, which enables modulating neural activity at inner, and at superficial, brain locations. This node and node 31 (US8523753 on magnetic stimulation to brain) linked MG2 to MG1 in the 2010s. Some of the other applications not covered by these two clusters are stimulation for pain management and migraine (nodes 15 and 23), tinnitus (node 21), facial nerve stimulation (node 34), and for improving blood circulation (node 25).

#### Main Paths for Microwave/RF Therapeutic Domain

**Figure 8:**
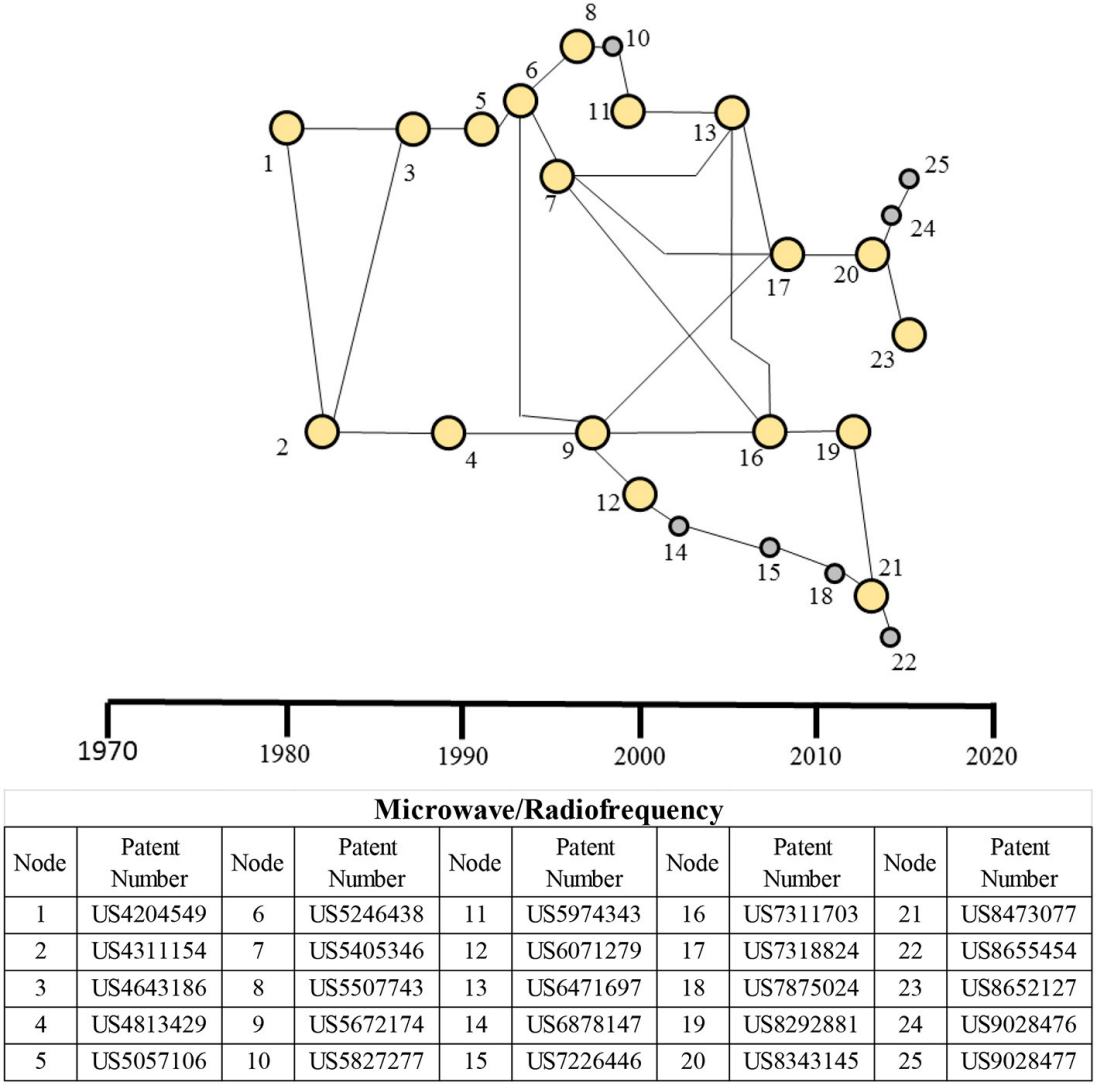
Main paths for the microwave/RF-based therapeutic domain. The table presents details of each patent in the main paths. High-persistence patents are shown as bigger yellow circles, whereas the low-persistence patents are shown as smaller gray circles.

The main paths for microwave/radiofrequency therapeutic domains are shown in Figure 8. In contrast to the other energy-based therapeutic domains, the microwave/RF domain shows only one cluster. Although a few patents—nodes 6, 8, and 9—are related to RF energy application, the remaining patents in the main paths are microwave patents, indicating how microwave rapidly took over the RF-based modality. However, nodes 6 and 9 based on RF are important patents in the advancement of MW-based therapeutic technology. Other important nodes are 7, 13, 16, 17, and 20, all using the microwave energy.

#### Main Paths for the Optical Therapeutic Domain

**Figure 9:**
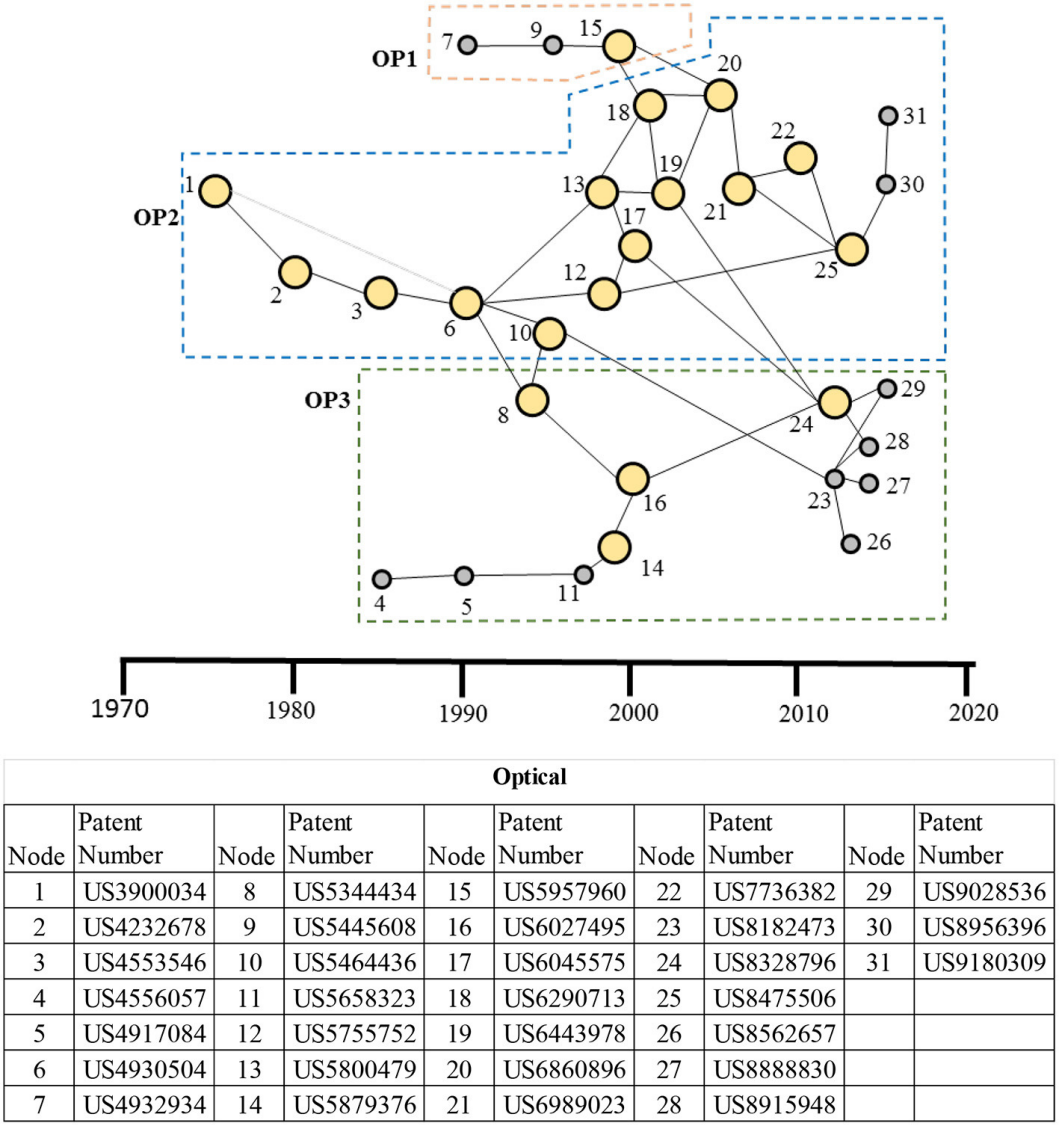
Main paths for the optical therapeutic domain. OP1: A patent cluster for photochemical stimulation of tissue; OP2: a patent cluster for stimulation of nerves of using light; and OP3: a patent cluster for skin treatment using a laser. The table presents details of each patent in the main paths. High-persistence patents are shown as bigger yellow circles, whereas the low-persistence patents are shown as smaller gray circles.

The main paths for the optical therapeutic domain are shown in Figure 9, in which three technological clusters have been identified. The patent cluster OP1, the smallest among the three, is related to photochemical stimulation of tissue, in which the photo-reactive agents are activated with light. The patent cluster OP2, the largest among the three, is related to the stimulation of nerves using light. The cluster had its beginnings around mid-1975. The early patents from the 1970s to the late 1990s (nodes 2, 3, 6, and 13) utilize infrared (IR) radiation for stimulating tissue, including nerves. The patents after the mid-2000s until recent ones (nodes 21, 22, 25, 30, and 31) are inventions for stimulating *nerve tissue* optically, including optical nerves. Although the OP1 cluster related to photochemical stimulation of tissue emerged later but has still contributed to the development of nerve stimulation with two patents in OP2—US6290713 (node 18) related to flexible illuminators and US6443978 (node 20) related to optical-stimulation of tissue—linking OP1 cluster. US8475506 (node 25) from Lockheed Martin is an additional key patent in this OP2 cluster for stimulation of nerve tissue and utilizes an array of two or more vertical-cavity surface-emitting lasers (VCSELs) to stimulate human tissue. The patent cluster OP3 is primarily concerned with the treatment of skin ailments such as vascular and pigmented lesions in the skin using laser energy. Although the OP3 cluster began independently in the 1980s, it had significant interaction with OP2 later in 1990s, leveraging the work of, and linking to, OP2 through US4556001 (node 8) on photodynamic therapy and much later on the photo-cosmetic device for skin and the light energy head with higher efficiency. The nodes 16 (US6027495 for diode laser treatment of vascular and pigmented skin lesions), 23 (US8182473 photo-cosmetic device with phase-change-based subsystem for cooling tissue), and 24 (US8328796 light energy head incorporating efficient means for minimizing backscattering) are additional key inventions in this main path for the OP3 cluster.

#### Main Paths for the Ultrasound Therapeutic Domain

**Figure 10:**
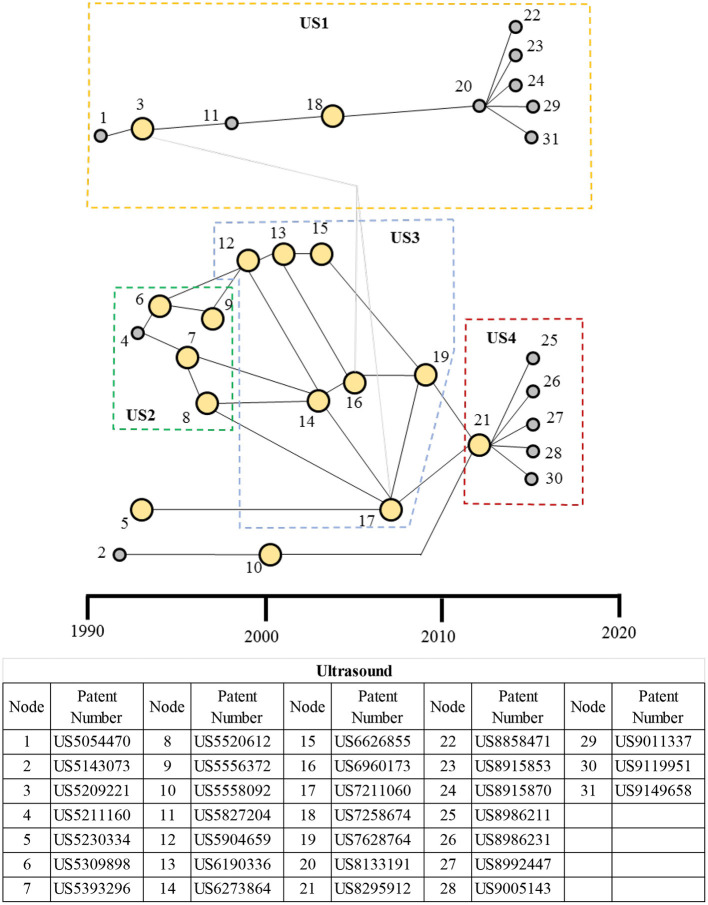
Main paths for the ultrasound therapeutic domain. US1: A patent cluster for tissue lesioning; US2: bone growth technologies; US3: a patent cluster for wound-healing technologies; and US4: a patent cluster for nerve-stimulation technologies. The table presents node numbers with corresponding patents and their description. High-persistence patents are shown as bigger yellow circles, whereas the low-persistence patents are shown as smaller gray circles.

The main paths for the ultrasound therapeutic domain are shown in Figure 10 with four patent clusters identified. Patents in ultrasound cluster 1 (US1) are related to tissue lesioning. For example, a high-persistent patent US7258674 by Liposonix (node 18 in US1 cluster) utilizes high-frequency ultrasound to break down fatty tissue. Several patents fan out of the patent labeled node 20 by Syneron Medical Ltd, out of which one (node 32) belongs to Syneron Medical; and, interestingly, the remaining seven patents related to different treatment applications all belong to Guided Therapy Systems, Inc. Patent cluster US2 includes patents related to non-invasive technologies for stimulating bone growth in fractures, using pulsed standing waves. This cluster gives rise to non-invasive wound (e.g., ulcers)-healing technologies in patent cluster US3, which, in turn, gives rise to the nerve-stimulating technologies in patent cluster US4. The patents labeled 14 (US6273864), 17 (US7211060) (which link US3–US2), and 19 (US7628764), all by Exogen Inc. are key technologies for portable ultrasound devices for promoting wound healing. US8295912 (node 21), which links US3 and US4 clusters, is a key technology from Kona Medical, Inc. for inhibiting nerves around arteries (especially those supplying the kidneys) and has given rise to additional eight patents in patent cluster US4; seven of which are by Kona Medical Inc. itself. Six of the eight patents further extend the work on stimulation of nerves surrounding blood vessels, and two patents (nodes 33 and 34) enhance the energy delivered to the nerve through the improved coupling of ultrasound energy sources to the tissue, using an agent and by using an intravascular catheter.

## Discussion

The specific inventions underlying energy-based therapeutics are quite broad and interactive with an even wider range of other technologies. This is seen in the main path results since this technique only considers a select set of high-persistence patents, which the technique identifies as having important longer-lasting effects on the development within the domain. Even with this much smaller set of patents, Figures 6–10 and reading of the key patents demonstrate wide interaction of each domain with the larger technological and scientific front. Although there is some overlap with the most highly central patents, these are expected to be different since the most central patents are those most important throughout the patent system throughout overall technological development. Such highly central patents are broad and well-connected throughout the entire patent network. However, high-persistence patents are well-connected in the domain and, therefore, better represent the most important patents and key technological events in the domain. Not surprisingly, patent clusters in the main paths can be linked to, and influence, therapy trends in various areas. We first discussed these main path clusters and their links with the therapies and followed by a discussion of improvement rates.

### Main Path Discussion

The main path analysis of the electrical therapeutic domain (Figure 6) shows that the E1 cluster related to cardiac stimulation technologies is the dominant application within this domain. This is consistent with our qualitative study of the domain and with our qualitative study of patents in this domain. The objective main path technique has been able to identify this dominance clearly showing the value of the method and support for wider application. Recently, the E2 cluster on neuromodulation, which has its roots in cardiac stimulation, has had the most active and experienced rapid growth. Although neuromodulation has received much attention over the last two decades (1, 5–8), the main path analysis demonstrates that neuromodulation technologies have been evolving from much earlier, starting as early as the 1980s. The cardiac stimulation patents have translated to therapies in the form of external and internal pacemakers (e.g., single chamber, dual chamber, biventricular) as treatments for arrhythmia, atrial fibrillation, bradycardia (slow heartbeat), and tachycardia (high heartbeat), and others. The cardiac-stimulation-leads-industry revenue is projected to grow to the multibillion dollar level with Aesculap, Biotronik, Estech, Medtronic, Oscor, Vitatron, BD, B. Braun, Edwards Lifesciences, BioTrace Medical, and Teleflex Inc. as the key players (76, 77). The E2 cluster on neuromodulation has been targeting an array of chronic diseases and disorders such as migraines, arthritis, asthma, Alzheimer's disease, depression, diabetics, and digestive disorders [2). Given the wide range of organs it innervates, the vagus nerve is a popular target for stimulation. Transcutaneous Gammacore stimulator, which stimulates the vagus nerve to provide relief from migraines, is an exemplar device (2). The electronic neuromodulation market is projected to grow to $16B by 2025 with Galvani Electronics and SetPoint Medical as key players in this market (78, 79).

The main path analysis shows that the magnetic therapeutic domain (Figure 7) was centered on stimulating bone and tissue growth (MG1 cluster) until the early 2000s. Since then, the focus has dramatically shifted to transcranial brain stimulation (MG2 cluster), a form of neuromodulation with an emphasis on brain stimulation. The non-invasive nature of the treatment makes it a particularly attractive modality, and its popularity for treating neurological (Parkinson's disease, epilepsy, essential tremor, and dystonia) and psychiatric disorders (depression, obsessive compulsive disorder) is growing (80–83). Some key players in transcranial brain stimulation are Abbott Laboratories, Medtronic PLC, Aleva Neurotherapeutics SA, Functional Neuromodulation, NeuroPace, Inc., Nevro Corporation, and Neuronetics Inc. (83).

The main path analysis of the microwave/RF therapeutic domain (Figure 8) has a single cluster and is used for ablation of tissue for the treatment of cancer (liver, kidney, prostate, lung, and bone metastasis) (84, 85), cardiovascular diseases (e.g., arrhythmias) (57, 86), gynecological [e.g., endometrial ablation to destroy the uterine lining (87), urological (88), and orthopedic disorders. Among these treatments, tumor ablation has the largest share (84). Although RF technologies originated first, with the precision of microwave technologies and their ability to produce higher temperatures, larger ablation volumes, and shorter ablation time, as well as their ability to pass through low-conductivity tissues (e.g., fat and bone), the microwave technologies have become increasingly popular in the recent decades. Key market players in this ablation market are Covidien PLC, Medtronic, Inc., Biosense Webster, Inc., St. Jude Medical, Inc., and Boston Scientific Corporation (89).

The main path analysis of the optical therapeutic domain (Figure 9) exhibits three clusters with the nerve stimulation cluster (OP2) dominating, while the skin treatment cluster (OP3) has shown increased activity recently. Although the optical nerve stimulation started in the mid-1970s, much of the activity in the cluster occurred after the mid-1990s. Optical energy stimulation has been proposed as an alternative to electrical stimulation of nerves, with the optical energy providing superior spatial resolution and contactless treatment (13, 61, 90–92). Treatment of auditory (e.g., hearing impairment), ophthalmological (e.g., retinal diseases), neurological, and psychiatric disorders (e.g., stroke, neurotrauma, neurodegeneration, and memory and mood disorders) are some key applications of the optical stimulation using infrared, laser, or in combination with chemical means, targeting cochlear and retinal nerves or the brain (13, 49, 50, 91). Cochlear implant of Lockheed Martin Aculight is an exemplar device for improving the quality of hearing for patients with an auditory impairment that stimulates the cochlea optically instead of by the traditional approach using electrical stimulation (93).

Main path analysis of ultrasound therapeutics shows four clusters (Figure 10), with the wound-healing technologies cluster US3 dominating for a decade starting from the late 1990s. More recently, the neurostimulation technologies cluster US4 has shown more activity. Interestingly, the clusters US2, US3, and US4 evolve sequentially, building on the previous clusters and focusing on increasingly more complex tissues, starting with hard tissue (e.g., for the treatment fractures in bones), soft tissue (e.g., for the treatment of wounds such as on skin and muscle), and then neural tissue. US1 cluster, independent from the other three and related to tissue lesioning, provides a non-invasive treatment, for example, for uterine fibroids (by ablating or shrinking), tremors and Parkinson's disease, and reduction of pain in bone cancer. Ultrasound lesioning is viewed as an alternative to microwave/RF ablation in some applications, and, in the case of tremors and Parkinson's disease, it competes with electrical deep brain stimulation (DBS) technologies. InsighteExBlate Body and ExBlate Neuro are exemplar systems that utilize high-frequency ultrasound for lesioning (94). Clinical studies have shown that low-frequency ultrasound stimulation is effective in treating bone fractures (related to the US1 cluster) and chronic wounds, such as pressure ulcers, diabetic wounds, and wounds in the lower extremity (related to the US2 cluster) (95–97). Sonic Accelerated Fracture Healing System (SAHFS) by Exogen, Inc. and MIST Therapy System 5.0 by Celleration, Inc. are ultrasound therapeutic devices indicated, respectively, for bone fracture and wound cleaning (98, 99). Transcranial-focused ultrasound (an example of US4) is emerging to be an alternative treatment approach for neuromodulation with non-invasiveness, spatial focus, and deep penetration (of the brain) to treat neurological (e.g., tremors, neuropathic pain, Alzheimer's, Parkinson's) and psychiatric disorders (e.g., depression, obsessive-compulsive disorder) and will likely compete with deep brain stimulation (DBS), using electrical leads (invasive), and transcranial magnetic stimulation (TMS) (with broad areas of action) (11, 12).

In summary, the discussion presented above demonstrates (1) that the clusters identified as important by the main path analysis are the ones that are significant therapeutically and (2) that neuromodulation is now the most important area in the electrical, magnetic, optical, and ultrasound areas. The microwave main path is the only one that exhibits a single group, and it is associated with ablation. These four domains active in neuromodulation appear to be producing competing and complementary therapeutics.

### Improvement Rates Discussion

In addition to the main path results, which, as just discussed, are found to be related to therapy trends, this research has provided estimates of performance improvement rates for the five energy-based stimulation domains and a comparable rate for pharmaceuticals. We note again that the high-centrality patents important in showing high rate domains are not the same as the high-persistence patents signaling important within-domain trends. In general, differences in performance improvement rates are the key factor in technological change, which occurs when a faster-improving technology achieves equal or better value in a given application than a more slowly improving incumbent technology (100). The improvement rates can also be understood from a theoretical point of view (101) as different because of two key factors—complexity or component interactions slow improvement rates while favorable scaling can accelerate improvement rates. In this section, we first attempt to explain the differences among the performance rates based on these fundamental factors. After considering the fundamental explanations, we discuss the potential practical significance of some of the rate differences we found.

The highest improvement rate identified in this study is for the microwave stimulation domain, the value of 88% improvement per year is higher than any other medical domain yet studied but is not as high as software-dominated domains identified by Benson and Magee (102) nor as high as many domains (mostly software related) in a recent study by Singh et al. (75). The key factor in achieving very high rates of improvement in numerous software domains is the low interaction (high modularity) among components that such systems exhibit; we believe that such low interactions are also the key reason for the higher rate for the microwave domain. The small area and close control of the stimulated area facilitate the low interaction (reduced side effects in the form of damage to the adjacent healthy tissue). The lowest estimated rate of improvement in this study was for the pharmaceutical domain, and the high interactivity of such therapies is well-known and consistent with the low estimate. Among the energy-based stimulation domains, we identified the magnetic domain as improving least rapidly. The large tissue areas stimulated by magnetic stimulation (11, 12) are the probable explanation for this domain lagging within the energy-based stimulation domains.

There are several indicated therapies change phenomena that are consistent with, and thus potentially explained by, the rate differences we have observed. We note that the techniques used in this research are reliable indicators of the rate of improvement of a technology domain and can indicate which technology area is likely to dominate in the future. However, they are not capable of defining future technological or therapeutic developments (for example, adoption, and dominance of a given technology) in any further detail as a clinical science and regulatory practice also have a strong influence on development dynamics. All the conclusions are, nonetheless, useful from a resource allocation point of view and thus are of value for strategists and policymakers. We now discuss four rate difference findings with strategic impact. First, the fastest improving domain (microwave) is the only main path with a singular cluster, and this is consistent with a fast-improving domain. Indeed, the initial patents within this main path show microwave technology quickly dominate other RF stimulation technologies as would be expected if microwaves were improving at a faster rate than competitors. A possible second therapy change could be if microwave stimulation begins to replace the other energy-based stimulation domains. However, it will only dominate other stimulation domains when and if it can be adapted for effective usage in other areas. It is not clear to us if this will be possible for cardiac or for nerve stimulation that is becoming the dominant application in other domains. However, if this can be done, microwave stimulation for cardiac and nerve stimulation could be a dominant future therapy.

The third practical implication of our observed improvement rates is that bone and tissue growth by magnetic stimulation (MG1 in the main path) did not continue beyond the early 2000s but ultrasound bone and wound healing did continue until around 2010. The higher improvement rate for ultrasound (42%) vs. magnetic (14%) is a likely explanation for this difference.

The fourth and somewhat speculative but potentially very important practical implication of our findings involves pharmaceuticals vs. energy-based therapies. The overall substantially higher rates of improvement for energy-based therapeutics relative to the currently dominant therapy of pharmaceuticals could foreshadow a major transition in medicine. This might particularly apply to developments in the fastest improving domain (microwave/RF) where a major application aim found in the main path was for cancer tumor removal (in this case, potentially displacing major surgery, and/or pharmaceuticals). However, the advent of energy-based therapies might well be in roles that enhance or modify pharmaceuticals rather than replace them. A study looking at such possibilities would be very valuable to those who care about large transitions in medical therapies.

We conclude with a discussion of a limitation of our study. Our study utilizes only patents granted by the USPTO. These include patents filed by US institutions as well as by international institutions. We believe that the patents filed at the USPTO by international institutions should be important as the applicants are going outside their native patent jurisdiction (e.g., Germany, Japan, the UK, etc.) for patent protection in the US at a considerable cost. Although the patent sets for the five energy-based therapeutic domains do not include *all* the patents across the globe, we reason that our patent sets include important patents globally and thus provide a sound basis for analysis and broad insights useful in the context of US and internationally.

## Data Availability Statement

The original contributions presented in the study are included in the article/Supplementary Material, further inquiries can be directed to the corresponding authors.

## Author Contributions

All authors listed have made a substantial, direct and intellectual contribution to the work, and approved it for publication.

## Conflict of Interest

The authors declare that the research was conducted in the absence of any commercial or financial relationships that could be construed as a potential conflict of interest. The reviewer HY declared a shared affiliation, with no collaboration, with the authors to the handling editor at the time of the review.

## Publisher's Note

All claims expressed in this article are solely those of the authors and do not necessarily represent those of their affiliated organizations, or those of the publisher, the editors and the reviewers. Any product that may be evaluated in this article, or claim that may be made by its manufacturer, is not guaranteed or endorsed by the publisher.

## Footnotes

^1^Note that the second most active assignee is a leading aerospace company. The patents resulted from an aborted diversification-by-research activity and were eventually sold to other participants.
