# Supplementary material for: Technological Improvement Rates and Evolution of Energy-Based Therapeutics
Source: Front Med Technol. 2021 Sep 3;3:714140. doi: 10.3389/fmedt.2021.714140 (PMC8757806; doi:10.3389/fmedt.2021.714140)
Supplement: Supplementary file 1 [file Table_1.DOCX]

**Patent set for Ultrasound energy-based therapeutic domain**

**(Granted between 1970-2015)**

US3828769 US3970076 US3958559 US4077401 US4078556 US4248232 US4535759 US4651716 US4820260 US4979501 US4955365 US4982730 US5012797 US5054470 US5018508 US5003965 US5040537 US5103806 US5158070 US5172692 US5143073 US5115805 US5143074 US5111822 US5143063 US5186162 US5230334 US5211160 US5209221 US5191880 US5231975 US5247935 US5323769 US5350351 US5307816 US5309898 US5316000 US5318570 US5307812 US5323779 US5393296 US5413550 US5409446 US5443069 US5472405 US5458130 US5456686 US5380411 US5545124 US5549544 US5529572 US5547459 US5501655 US5573497 US5558623 US5520612 US5496256 US5509896 US5524624 US5556372 US5558092 US5485839 US5656015 US5626554 US5664570 US5688235 US5665054 US5595178 US5643179 US5601526 US5692509 US5624382 US5687729 US5636632 US5762616 US5720710 US5827204 US5738635 US5727558 US5792078 US5752924 US5730705 US5727556 US5759162 US5904659 US5984881 US5989202 US5873844 US5984882 US5947901 US5931783 US5938600 US6113558 US6086535 US6165144 US6022317 US6071238 US6039689 US6113570 US6068596 US6164283 US6012457 US6273864 US6190336 US6325769 US6251088 US6206843 US6231528 US6280402 US6267734 US6261249 US6322527 US6217530 US6183426 US6321109 US6264608 US6361510 US6394969 US6355006 US6443914 US6383151 US6413230 US6368292 US6406443 US6361509 US6350245 US6488639 US6436061 US6436060 US6432070 US6379320 US6432067 US6450979 US6454730 US6478754 US6340352 US6500141 US6413254 US6409720 US6500133 US6451013 US6428478 US6432069 US6374132 US6498945 US6490469 US6461314 US6428477 US6524261 US6669655 US6565520 US6626854 US6514220 US6645162 US6543272 US6623430 US6508774 US6506171 US6607498 US6626855 US6613004 US6666834 US6533726 US6666835 US6569170 US6652473 US6613005 US6582381 US6589174 US6569099 US6589191 US6656136 US6599256 US6508783 US6511428 US6535761 US6635017 US6533803 US6685656 US6793635 US6692450 US6733450 US6778848 US6790187 US6679855 US6773408 US6736784 US6770031 US6716168 US6726627 US6823216 US6676601 US6755796 US6740040 US6736835 US6682502 US6687537 US6689128 US6719694 US6953438 US6960173 US6964647 US6887239 US6936046 US6953460 US6916296 US6929608 US7125387 US7128719 US7108663 US6981952 US7025735 US7090649 US7211054 US7189209 US7241270 US7311677 US7220232 US7229423 US7294125 US7305264 US7175599 US7211055 US7273458 US7283861 US7165451 US7223239 US7211060 US7311678 US7258674 US7416535 US7429248 US7413552 US7350522 US7344509 US7429249 US7399284 US7377905 US7410469 US7347855 US7335169 US7331951 US7628764 US7559905 US7637877 US7553284 US7500956 US7481781 US7507212 US7615015 US7494467 US7497119 US7645244 US7806839 US7789841 US7828754 US7662114 US7771359 US7699780 US7819826 US7699778 US7662098 US7742804 US7686763 US7841984 US7815570 US7819007 US7942835 US8043234 US7909782 US7985189 US7981060 US8048006 US7998095 US7993331 US8079966 US8057409 US8040756 US7914469 US7972286 US7993289 US7883481 US7967764 US8034004 US7875023 US8002706 US7914470 US7892191 US8226582 US8221338 US8162859 US8251929 US8262591 US8123707 US8133191 US8295912 US8292834 US8235919 US8337432 US8092401 US8224423 US8105248 US8337407 US8088073 US8333721 US8460221 US8556813 US8353834 US8500641 US8535250 US8454540 US8617145 US8603086 US8740821 US8808204 US8764687 US8801615 US8858440 US8882792 US8915949 US8905949 US8840556 US8696612 US8845558 US8892200 US8905935 US8727987 US8845559 US8870796 US8852103 US8897871 US8790359 US8831708 US8915853 US8858471 US8886304 US8790281 US8911346 US8915870 US8864744 US8926533 US8961441 US8932238 US8979775 US9147046 US8977354 US9180315 US9119954 US9050448 US9011337 US9180314 US9132287 US9114247 US9084878 US9144694 US9028434 US8972019 US9039617 US9022935 US9199096 US9114240 US8956277 US9119951 US8968171 US9114245 US9044598 US9199097 US9101752 US9061131 US8992958 US8968205 US8966981 US9149658 US9039624 US8932239 US9184373 US9107798 US8961391 US8974446 US8986231 US8992447 US8945015 US9005143 US8929979 US9037247 US9174065 US8986211 US9005144 US8942781 US9005100
