# Supplementary material for: Technological Improvement Rates and Evolution of Energy-Based Therapeutics
Source: Front Med Technol. 2021 Sep 3;3:714140. doi: 10.3389/fmedt.2021.714140 (PMC8757806; doi:10.3389/fmedt.2021.714140)
Supplement: Supplementary file 2 [file Table_2.DOCX]

**Patent set for Electrical energy-based therapeutics domain.**

**(Granted between 1970-2015)**

USR26810 US3516412 US3543761 USR26809 US3590822 US3563245 US3572345 US3596662 US3572344 US3568660 US3664347 US3650276 US3654933 US3640284 US3650275 US3719190 USR27569 US3774618 US3738368 US3754555 US3749101 US3724467 US3737579 US3738370 US3769984 US3749100 US3757789 US3760812 US3731376 US3729008 US3752939 US3814104 US3804098 US3844292 US3857399 US3800800 US3788329 US3835864 US3825015 US3815611 US3837347 US3880169 US3902501 US3865118 US3875947 US3915174 US3866615 US3893461 US3906960 US3903897 US3903896 US3924639 US3866613 US3908668 US3898994 US3918440 US3994302 US3976082 US3981309 US3949757 US3978865 US3937225 US3935864 US3939843 US3974834 US3977411 US3943936 US3943938 US3999555 US3933147 US3995644 US3943932 US3957056 US3964473 US3995623 US4027677 US4026303 US4044774 US4010758 US4026302 US4010755 US4057067 US4033357 US4058128 US4026301 US4030509 US4052991 US4030508 US4011875 US4010757 US4046151 US4010756 US4026300 US4033355 US4011861 US4000745 US4058116 US4052754 US4125116 US4112952 US4066085 US4106512 US4106511 US4124028 US4103690 US4101984 US4088138 US4146035 US4136701 US4146037 US4146036 US4177818 US4142530 US4136703 US4144890 US4169479 US4161952 US4154247 US4144889 US4136702 US4156429 US4149542 US4162679 US4142531 US4135518 US4153060 US4175565 US4209019 US4235246 US4236529 US4198991 US4187853 US4207903 US4217913 US4214594 US4233992 USR30366 US4214804 US4198963 US4299239 US4257428 US4280512 US4262678 US4282886 US4257429 US4280510 US4270549 US4258724 US4282885 US4285347 US4269198 US4287896 US4301815 US4271846 US4281668 US4280511 US4280513 US4280503 US4289144 US4291707 US4243050 US4296760 US4273137 US4258725 US4284085 US4261372 US4280514 US4256115 US4271847 US4244373 US4245643 US4281669 US4279256 US4284856 US4267838 US4306560 US4262672 US4262982 US4323081 US4357947 US4317459 US4357946 US4325389 US4328813 US4355646 US4341226 US4327747 US4350169 US4311153 US4351345 US4360031 US4332259 US4352360 US4328812 US4336811 US4365639 US4354508 US4317458 US4351330 US4357497 US4314095 US4345606 US4401127 US4402330 US4413636 US4401126 US4407303 US4374527 US4394866 US4381014 US4414986 US4379462 US4409994 US4386615 US4422460 US4381013 US4369791 US4402328 US4402329 US4407302 US4383532 US4408604 US4388930 US4378023 US4418704 US4393883 US4387717 US4387727 US4407304 US4458695 US4444206 US4475560 US4465079 US4437475 US4444207 US4452254 US4424818 US4454888 US4440178 US4442840 US4479500 US4488561 US4463765 US4458677 US4461300 US4467817 US4442846 US4487210 US4430999 US4481953 US4432377 US4444195 US4450846 US4485813 US4466690 US4462402 US4462401 US4437474 US4484586 US4552157 US4499907 US4530368 US4541440 US4313448 US4553554 US4516584 US4493329 US4550737 US4498482 US4519404 US4549556 US4534366 US4506679 US4538623 US4497326 US4512351 US4509519 US4502492 US4506674 US4548203 US4542752 US4506680 US4549557 US4519403 US4532931 US4522212 US4559951 US4519400 US4515167 US4514589 US4567901 US4628944 US4624266 US4585013 US4564024 US4608985 US4574814 US4592372 US4567900 US4582069 US4608986 US4573481 US4611604 US4570642 US4590949 US4580578 US4628942 US4628943 US4624265 USR32227 US4602645 US4603704 US4566467 US4630617 US4602624 US4574807 US4602637 US4564023 US4577643 US4572214 US4603696 US4614192 US4627439 US4620550 US4602638 US4603705 US4590950 US4590946 US4612940 US4600017 US4577642 US4573480 US4622975 US4677989 US4669488 US4677990 US4646755 US4643201 US4649937 US4667686 US4637404 US4649938 US4699157 US4649936 US4643202 US4679572 US4662382 US4683890 US4637405 US4708145 US4662377 US4641656 US3942536 US4690143 USH356 US4708143 US4651751 US4649935 US4633889 US4641664 US4664120 US4658835 US4640298 US4706682 US4672979 US4693258 US4706688 US4683896 US4706681 US4660571 US4721118 US4762136 US4784161 US4716888 US4722353 US4735205 US4774952 US4759378 US4773433 US4735206 US4760852 US4736747 US4730619 US4762135 US4716887 US4736752 US4765322 US4732161 US4765341 US4784159 US4784160 US4791939 US4774967 US4782818 US4769006 US4730389 US4785828 US4768523 US3911928 US4744371 US4744370 US4776349 US4883070 US4886074 US4800898 US4817634 US4844099 US4796643 US4858623 US4841971 US4809713 US4819662 US4865037 US4817608 US4860769 US4799499 US4825871 US4840186 US4832051 US4802481 US4834100 US4821723 US4809712 US4819661 US4798206 US4860446 US4848352 US4882777 US4817611 US4886075 US4852573 US4827932 US4807629 US4813421 US4873996 US4832048 US4870967 US4837049 US4827940 US4862887 US4850359 US4940065 US4971070 US4945922 US4913164 US4924881 US4953564 US4967766 US4974588 US4957118 US4938231 US4972833 US4944301 US4920979 US4919135 US4892108 US4922927 US4928689 US4913147 US4972847 US4974917 US4947866 US4917106 US4898183 US4961434 US4960134 US4924880 US4969463 US4941472 US4932407 US4919147 US4951687 US4972848 US4920978 US4930521 US4892102 US4960133 US4934381 US4909263 US4962767 US4911178 US4917104 US4979511 US4922912 US4967755 US4972846 US4940064 US4920980 US4954105 US4974595 US4979510 US4964414 US4922926 US4936317 US4969468 US4945342 US4976710 US4934367 US5014721 US5042463 US5020544 US5076285 US5016645 US5044374 US5014720 US5007435 US5016646 US5003992 US5020545 US5014696 US5007436 US5056517 US4989617 US5046511 US5052407 US5056516 US5052390 US5050601 US5010895 US4995389 US5002067 US5031618 US4991578 US4991603 US5036862 US5010894 US5033477 US5074313 US5040544 US5002053 US4998975 US5005587 US5009229 US5044375 US5000179 US5056532 US5038797 US5056531 US5060649 US5040545 US5000194 US5036854 US4988347 US5013310 US5036848 US5029585 US5058586 US5016808 US5069215 US5061282 US5022396 US5103837 US5097843 US5121754 US5127403 US5127421 US5170802 US5115818 US5170803 US5090422 US5111830 US5133365 US5111811 US5158097 US5152299 US5154183 US5154387 US5174288 US5099838 US5129405 US5086773 US5111812 US5117840 US5174289 US5123422 US5143090 US5129404 US5144960 US5099839 US5095902 US5087243 US5154182 US5154169 US5095916 US5139033 US5172694 US5105826 US5109842 US5143089 US5107856 US5086787 US5133353 US5095903 US5081990 US5095905 US5085218 US5119832 US5109844 US5152298 US5109851 US5170787 US5143067 US5154165 US5144952 US5117828 US5174303 US5156151 US5251643 US5181526 US5261417 US5263977 US5191901 US5224491 US5271417 US5261419 US5259394 US5259395 US5249574 US5238007 US5190052 US5179962 US5222506 US5269319 US5255691 US5265623 US5188104 US5241957 US5257634 US5176135 US5265602 US5263480 US5230337 US5199430 US5217027 US5271392 US5255693 US5247945 US5199443 US5267560 US5207218 US5269303 US5191885 US5269304 US5261418 US5181511 US5235978 US5246014 US5217028 US5243977 US5213113 US5228455 US5265608 US5215089 US5224477 US5178149 US5269810 US5190053 US5255692 US5231996 US5199442 US5203348 US5251634 US5178161 US5197491 US5273053 US5231995 US5267564 US5215088 US5238006 US5239999 US5269326 US5199433 US5243980 US5255679 US5207683 US5263493 US5257635 US5230349 US5228442 US5242441 US5179952 US5222501 US5201903 US5354328 US5324324 US5300108 US5304218 US5336254 US5327909 US5330525 US5336253 US5358517 US5282845 US5324325 US5374285 US5354327 US5376108 US5344439 US5350419 US5282844 US5344438 US5370679 US5275171 US5330524 US5330522 US5279291 US5300107 US5281219 US5314461 US5300110 US5314463 US5324321 US5330523 US5366493 US5342414 US5314459 US5304139 US5282837 US5314460 US5370671 US5318572 US5342409 US5366496 USR34502 US5314462 US5376103 US5312439 US5306292 US5324328 US5330520 US5325870 US5358516 US5330515 US5292344 US5332401 US5360440 US5342407 US5356427 US5304208 US5336252 US5374287 US5350404 US5360442 US5370665 US5324327 US5366494 US5360441 US5374279 US5370678 US5314464 US5330521 US5282468 US5324326 US5348554 US5334193 US5374286 US5314495 US5366489 US5279299 US5324311 US5324322 US5361760 US5375594 US5304219 US5376109 US5342413 US5313943 US5284161 US5336251 US5300106 US5331959 US5365926 US5354297 US5303704 US5309910 US5343860 US5327905 US4711251 US5306236 US5327889 US5345936 US5323781 US5385579 US5447534 US5443491 US5387234 US5383924 US5476497 US5476496 US5411545 US5456709 US5433744 US5397343 US5476498 US5433742 US5397342 US5423883 US5464448 US5387232 US5423881 US5447533 US5423880 US5425756 US5411544 US5403354 US5385578 US5423879 US5454839 US5456708 US5476503 US5443492 US5431696 US5423872 US5387233 US5458632 US5466255 US5411547 US5423882 US5385577 US5423884 US5456707 US5431683 US5423865 US5466253 US5425752 US5476502 US5449381 US5411546 US5470346 US5433730 US5405375 US5405374 US5423878 US5466254 US5441527 US5411540 US5411527 US5383914 US5464431 US5439485 US5383922 US5383923 US5405373 US5405372 US5423876 US5431681 US5403353 US5447521 US5433729 US5454837 US5411535 US5466247 US5425755 US5423877 US5439484 US5458630 US5443493 US5476500 US5458629 US5476493 US5456699 US5456705 US5476494 US5423811 US5385574 US5464446 US5405376 US5398683 US5425751 US5464447 US5397339 US5400784 US5391200 US5397341 US5473812 US5476501 US5456706 US5447529 US5476499 US5450845 US5405362 US5429130 US5438987 US5429131 US5456254 US5466252 US5378177 US5472438 US5476495 US5397344 US5394880 US5415166 US5425364 US5417208 US5458631 US5465717 US5409008 US5409000 US5400783 US5417653 US5450846 US5385146 US5409652 US5406946 US5411025 US5545207 US5514173 US5571160 US5562722 US5578068 US5584874 US5531780 US5562723 US5522874 US5509924 US5575814 US5571157 US5489294 US5522872 US5531783 US5507787 US5545206 US5556425 US5545201 US5531778 US5545203 US5571162 US5514174 US5545205 US5571159 US5496362 US5522876 US5531782 US5487758 US5571158 US5522875 US5531781 US5531779 US5490862 US5540730 US5584873 US5534022 US5578069 US5486202 US5554178 US5571164 US5501703 US5562717 US5571156 US5571163 US5551427 US5562707 US5571118 US5554179 US5554176 US5562721 US5545183 US5480416 US5578067 US5507782 US5545202 US5522873 US5514172 US5486160 US5496360 US5575813 US5480421 US5545161 US5549653 US5509925 US5545204 US5487756 US5480419 US5542173 US5527358 US5496363 US5531776 US5507779 US5549615 US5487391 US5556424 US5545200 US5500011 US5492119 US5505201 US5480420 US5499981 US5507802 US5514171 US5565005 US5549108 US5578084 US5569883 US5485849 US5490323 US5522877 US5545219 US5584830 US5564440 US5555883 US5556423 US5487757 US5558073 US5579764 US5507303 US5551426 US5515848 US5582609 US5488768 US5516396 US5509419 US5557210 US5575788 US5517989 US5683444 US5658326 US5609622 US5658327 US5702437 US5643338 US5697964 US5683443 US5649976 US5628778 US5674274 US5628779 US5593433 US5683446 US5649975 US5683447 US5662698 US5645580 US5697965 US5641326 US5649970 US5683445 US5674271 US5628774 US5649967 US5601607 US5697966 US5662697 US5653734 US5662699 US5630839 US5314465 US5609611 US5632267 US5609621 US5702428 US5618287 US5702429 US5674259 US5603732 US5632770 US5626618 US5643330 US5674272 US5603730 US5674273 US5643328 US5653742 US5630838 US5645577 US5676694 US5693081 US5620451 US5634462 US5649974 US5609623 US5628775 US5697927 US5658278 US5702430 US5690648 US5702438 US5607422 US5683442 US5622168 US5649969 US5645585 US5639276 US5651767 US5609151 US5673704 US5630426 US5689877 US5687723 US5692926 US5683403 US5632085 US5628317 US5697975 US5632749 US5697804 US5679022 US5628780 US5642736 US5640955 US5628773 US5693074 US5690611 US5669790 US5674196 US5667615 US5595183 US5628313 US5741321 US5755759 US5755758 US5837007 US5733322 US5800497 US5833715 US5772693 US5782901 US5716391 US5776178 US5713945 US5755764 US5755767 US5851227 US5849032 US5755766 US5782898 US5837006 US5800496 US5824032 US5769881 US5824027 US5725566 US5796044 US5735891 US5800499 US5759202 US5800495 US5769077 US5803928 US5807399 US5733323 US5849033 US5843149 US5810887 US5853425 US5800498 US5713944 US5755763 US5716392 US5713922 US5846239 US5853424 US5836990 US5728149 US5741322 US5843141 US5800502 US5755765 US5716390 US5766224 US5800500 US5728140 US5800468 US5776179 US5792208 US5713926 US5824031 US5814092 US5843135 US5824016 US5782902 US5833714 US5755750 US5851226 US5836975 USR35779 US5814085 US5755761 US5707399 US5824028 US5800474 US5755760 US5755664 US5800501 US5713943 US5755762 US5849031 US5792194 US5824030 US5741319 US5785059 US5759471 US5782900 US5800465 US5800536 US5782873 US5749914 US5735885 US5722403 US5826576 US5843146 US5782892 US5720775 US5785040 US5814076 US5800482 US5759197 US5843150 US5709644 US5830210 US5843153 US5824026 US5843147 US5830213 US5853411 US5797902 US5843132 US5725524 US5766231 US5792067 US5728144 US5728148 US5836947 US5746722 US5843148 US5853373 US5730127 US5762583 US5824029 US5846238 US5772590 US5718701 US5792217 US5752915 US5716389 US5845396 US5823955 US5853368 USR35924 US5782239 US5730128 US5803083 US5732698 US5842984 US5722401 US5766042 US5814029 US5715818 US5713847 US5810725 US5715817 US5853409 US5836875 US5807144 US5738683 US5833673 US5722400 US5722416 US5718241 US5746616 US5779632 US5823956 US5769846 US5836996 US5797905 US5741214 US5722402 US5843093 US5810802 US5871529 US5957965 US5897585 US5983142 US5968086 US5922014 US5964795 US5928276 US5928278 US5876430 US5931864 US5928277 US5957970 US5951597 US5954759 US5999858 US5861023 US5931863 US5865843 US5948015 US5902330 US5913887 US5876431 US5948014 US5978714 US5925073 US5871531 US5991667 US6006137 US5957968 US5987361 US5919220 US5876429 US5897586 US5899933 US5964794 US5968085 US5991668 US6002964 US5928269 US5916243 US5935160 US5871530 US5922015 US5897583 US5871532 US5999859 US5919222 US6006138 US5904711 US5978705 US5935159 US5931862 US5957966 US5871533 US5865842 US5873896 US5957967 US6002969 US5871507 US5954754 US5902329 US5916238 US5954760 US5908447 US5935155 US5925070 US5968082 US5935158 US5957958 US5938689 US5913882 US5925069 US5948007 US5895415 US5964793 US5871534 US5995876 US6006139 US5968087 US5871528 US6006122 US5954757 US5891137 US5997536 US5954761 US5897584 US6006130 US5871483 US5871525 US6006134 US5987746 US5876443 US5895417 US6001095 US5938596 US5902331 US5881731 US5875778 US5964702 US5869804 US5895416 US6002955 US5879295 US5897554 US6006124 US5902236 US5861024 US5910120 US5863291 US5922017 US5906613 US6002968 US5891135 US5891136 US5971983 US5992418 US5891138 USR36120 US5919221 US5967977 US5899875 US5980515 US5913854 US5899847 US5951471 US5938694 US5925038 US5928159 US5987344 US5941904 US6004262 US5881727 US5885278 US5991649 US5919188 US5882346 US5860920 US5921924 US5876336 US5860974 US5904651 US5873901 US5916158 US5904680 US5855552 US5954665 US6006755 US5865839 US5986034 US5908385 US6104961 US6066165 US6055456 US6163727 US6066166 US6157862 US6038482 US6108582 US6151526 US6152954 US6021355 US6078839 US6104960 US6148238 US6078841 US6125302 US6061598 US6119042 US6119044 US6129753 US6163729 US6144882 US6070104 US6129752 US6052625 US6021354 US6136021 US6055457 US6129751 US6058332 US6129750 US6132456 US6104957 US6134478 US6047217 US6097986 US6085119 US6078840 US6011996 US6085117 US6010526 US6038483 US6026332 US6032079 US6152955 US6041256 US6083247 USH1905 US6018683 US6070105 US6076019 US6144883 US6163728 US6094596 US6112124 US6041258 US6167314 US6035239 US6141593 US6081745 US6151527 US6038484 US6047210 US6134477 US6122552 US6148237 US6096064 US6157860 US6083252 US6038463 US6018682 US6038472 US6122548 US6026326 US6146380 US6068650 US6144879 US6096069 US6032074 US6129749 US6016436 US6161047 US6094598 US6167315 US6029091 US6091978 US6149657 US6018684 US6141594 US6049736 US6142941 US6032061 US6086549 US6032077 US6090104 US6038480 US6083216 US6026567 US6142994 US6119041 US6144870 US6052608 US6044304 US6033414 US6064902 US6125290 US6091995 US6096035 US6042580 US6063077 US6109269 US6132390 US6136005 US6044302 US6074422 US6128537 US6032062 US6015407 US6033402 US6071282 US6159163 US6161029 US6086582 US6165180 US6014579 US6014584 US6055453 US6035226 US6120500 US6099526 US6035224 US6061595 US6167291 US6144866 US6086581 US6152882 US6092528 US6019877 US6148222 US6146379 US6156018 US6129685 US6056744 US6151519 US6099524 US6024743 US6034295 US6149678 US6146381 US6070094 US6024764 US6052607 US6110100 US6096036 US6163716 US6120502 US6129724 US6030382 US6106522 US6188932 US6259954 US6230061 US6330481 US6181972 US6298272 US6173206 US6219582 US6178356 US6269272 US6188931 US6219581 US6212434 US6175769 US6249708 US6301507 US6321125 US6263249 US6309410 US6195586 US6192280 US6236892 US6249709 US6292704 US6253110 US6256541 US6173205 US6181971 US6240322 US6236893 US6240320 US6304786 US6256542 US6208881 US6201994 US6205361 US6324415 US6253111 US6240321 US6238429 US6289251 US6330480 US6289250 US6263250 US6295476 US6308103 US6321124 US6321123 US6295475 US6256543 US6185463 US6321122 US6198973 US6246915 US6181973 US6178355 US6285910 US6266568 US6223087 US6259953 US6192279 US6212435 US6324433 US6304787 US6278897 US6292699 US6292701 US6327498 US6292702 US6230052 US6292703 US6169924 US6238423 US6249707 US6292695 US6280462 USR37463 US6321102 US6295474 US6308105 US6280463 US6282445 US6269270 US6304777 US6193743 US6266564 US6298270 US6230057 US6304785 US6178354 US6298268 US6315781 US6321104 US6208882 US6185464 US6178357 US6243603 US6308104 US6266567 US6278896 US6289247 US6181961 US6259951 US6169922 US6263237 US6214002 US6210417 US6233491 US6324429 US6314324 US6324434 US6301500 US6176242 US6324435 US6216045 US6306168 US6241724 US6254425 US6318374 US6319241 US6302880 US6265691 US6325764 US6181964 US6241701 US6261224 US6185465 US6256540 US6227203 US6179835 US6169916 US6263224 US6216043 US6304784 US6309370 US6254598 US6221070 US6308091 US6308090 US6312425 US6309385 US6270496 US6185452 US6233482 US6289894 US6217369 US6171306 US6315776 US6198974 US6293594 US6197021 US6477429 US6366819 US6397109 US6370434 US6381500 US6434431 US6501993 US6487453 US6400992 US6501994 US6490489 US6501992 US6377856 US6374141 US6397110 US6418348 US6456888 US6405091 US6477427 US6389320 US6360130 US6445958 US6363287 US6363286 US6463333 US6459937 US6363288 US6438426 US6438427 US6345204 US6493590 US6400976 US6498954 US6440488 US6438425 US6434430 US6477428 US6345198 US6366820 US6438421 US6408213 US6356791 US6501991 US6456890 US6374142 US6445954 US6438416 US6456889 US6438423 US6356787 US6493591 US6343226 US6473654 US6430448 US6366815 US6477423 US6430442 US6347250 US6493592 US6339723 US6421567 US6501990 US6381495 US6385492 US6480747 US6430449 US6370427 US6466824 US6377853 US6442431 US6408214 US6385491 US6442435 US6411855 US6463334 US6337997 US6442424 US6341234 US6415184 US6484057 US6377857 US6393325 US6397108 US6430447 US6356786 US6360129 US6366808 US6421569 US6415187 US6389317 US6456866 US6473633 US6498943 US6353762 US6470219 US6453205 US6374143 US6473653 US6336047 US6425895 US6358256 US6489562 US6356792 US6342035 US6430426 US6373024 US6355064 US6463335 US6356777 US6447507 US6360750 US6430425 US6464700 US6478776 US6416510 US6358247 US6370429 US6408855 US6393327 US6449507 US6388866 US6427087 US6370435 US6475213 US6428536 US6482182 US6456886 US6379352 US6395016 US6443974 US6456864 US6353751 US6419674 US6356779 US6370411 US6428484 US6437075 US6405732 US6484054 US6437076 US6471699 US6500172 US6611721 US6606521 US6510348 US6553265 US6556873 US6549812 US6574512 US6600956 US6505082 US6654644 US6546293 US6580949 US6584362 US6567704 US6584363 US6574514 US6516230 US6549813 US6526321 US6510347 US6643552 US6516232 US6634364 US6587734 US6662055 US6633780 US6577904 US6505075 US6662045 US6625495 US6544270 US6549811 US6647291 US6606522 US6671553 US6671562 US6564101 US6539260 US6618627 US6658289 US6597953 US6549814 US6650945 US6564094 US6618623 US6560488 US6587719 US6556874 US6529778 US6512959 US6516231 US6628991 US6553266 US6560491 US6522927 US6529777 US6658295 US6671550 US6671561 US6542776 US6671560 US6553264 US6611710 US6671544 US6654638 US6636770 US6615085 US6658299 US6505081 US6571131 US6625496 US6542780 US6529776 US6600953 US6662053 US6650921 US6505078 US6577900 US6535762 US6539264 US6549810 US6587733 US6567705 US6606523 US6643550 US6522932 US6658302 US6611716 US6540742 US6546292 US6564107 US6574513 US6532388 US6532378 US6609032 US6571130 US6622051 US6510332 US6567699 US6542774 US6622046 US6650942 US6535764 US6512957 US6516227 US6665563 US6529774 US6544262 US6532390 US6556869 US6575894 US6584351 US6591143 US6666864 US6597955 US6505401 US6530954 US6547787 US6516226 US6603654 US6564106 US6620156 US6615483 US6610081 US6654634 US6529779 US6540765 US6671554 US6565503 US6647297 US6516223 US6659936 US6591144 US6652517 US6549797 US6671534 US6557559 US6658297 US6626841 US6512958 US6592581 US6582425 US6616657 US6666826 US6587731 US6669693 US6564078 US6522913 US6662035 US6658279 US6526302 US6654643 US6613047 US6511477 US6605089 US6669687 US6516808 US6540743 US6745079 US6819959 US6718211 US6687550 US6721604 US6801809 US6785576 US6792317 US6704604 US6711443 US6741893 US6684109 US6728579 US6763270 US6772015 US6714823 US6697677 US6813521 US6718212 US6799076 US6704605 US6748277 US6792318 US6697675 US6754539 US6684104 US6687542 US6687549 US6701191 US6766203 US6807447 US6721598 US6785577 US6741894 US6804553 US6745081 US6804561 US6754536 US6748268 US6738674 US6694190 US6754537 US6788975 US6738672 US6829508 US6778858 US6714822 US6708066 US6792314 US6735472 US6718207 US6760628 US6795736 US6718203 US6763268 US6799069 US6819954 US6792316 US6738673 US6760626 USR38654 US6823217 US6678564 US6832115 US6819957 US6718209 US6697676 US6675049 US6735471 US6757566 US6738663 US6778856 US6718208 US6772013 US6745082 US6757970 US6801805 US6786905 US6745075 US6829498 US6725096 US6836687 US6772014 US6792309 US6814733 US6748653 US6782619 US6795737 US6793670 US6748275 US6829509 US6711444 US6814732 US6836688 US6728563 US6687548 US6819958 US6714806 US6834200 US6736811 US6778861 US6733500 US6702777 US6701172 US6795733 US6743227 US6741878 US6795721 US6804545 US6755530 US6714809 US6673070 US6712814 US6882887 US6907296 US6944507 US6909918 US6882886 US6859667 US6952616 US6876885 US6871101 US6941174 US6968237 US6961621 US6868291 US6970748 US6889094 US6889091 US6931285 US6978185 US6889092 US6842648 US6973351 US6842649 US6925334 US6901297 US6909920 US6970746 US6968236 US6876887 US6909919 US6944506 US6968238 US6847845 US6928326 US6895283 US6970747 US6944505 US6920359 US6895278 US6934589 US6952613 US6901289 US6937897 US6901288 US6934583 US6980858 US6928312 US6868287 US6889093 US6907293 US6937891 US6920358 US6859666 US6950709 US6871091 US6973352 US6937907 US6973349 US6959215 US6975907 US6912425 US6931286 US6978180 US6885888 US6915169 US6912424 US6895279 US6980865 US6950710 US6922588 US6847849 US6850805 US6968234 US6978184 US6901287 US6925333 US6920361 US6895277 USR38705 US6980863 US6908470 US6907299 US6950707 US6892095 US6907298 US6901290 US6978178 US6904324 US6907295 US6954674 US6912423 US6893439 US6968235 US6937892 US6941169 US6912419 US6895280 US6973347 US6871085 US6980866 US6915165 US6915168 US6879860 US6971393 US6936040 US6947782 US6944489 US6862805 US6949763 US6939349 US6974457 US6970745 US6856840 US6855116 US6845266 US6904315 US6973346 US6966322 US6957103 US6976998 US6973340 US6944502 US6976967 US6876886 US6878118 US6955657 US6961602 US6978176 US6979319 US6913478 US7099718 US6985775 US7047082 US7146224 US7139611 US7020529 US6999819 US7082335 US7027876 US7107105 US7010358 US7079902 US7127301 US7155292 US7013182 US7146221 US7096071 US7139614 US7031777 US7110828 US7120504 US7127302 US7054692 US7155294 US7107104 US7047086 US7006875 US6988007 US7072719 US7058454 US7082337 US6985776 US6999821 US7069083 US7142914 US7149586 US7039470 US7085605 US6999820 US7065411 US7020518 US7010356 US7016736 US7076292 US7146222 US7155293 US7047081 US7092764 US7149588 US7146226 US6985777 US7076309 US7146227 US6988006 US7113828 US7146217 US7085602 US7058456 US7146225 US7127295 US7089065 US7031776 US6988001 US7107097 US7120503 US7120502 US7082336 US7142926 US7142919 US6985774 US7047074 US7062324 US7027874 US7047083 US7050856 US7010351 US6999814 US7013180 US7127291 US7043299 US7142928 US7117039 US7010357 US7013179 US6993384 US7079903 US7117033 US7065410 US7020527 US7120499 US7082332 US6993391 US7003350 US7047071 US7130699 US7013181 US7085606 US7110827 US7092765 US7041099 US7139612 US6993387 US7006873 US7118555 US7155276 US7089046 US7151965 US7006859 US7035680 US7024247 US7058455 US7103416 US7146220 US7120489 US7072720 US7117035 US6993395 US7035688 US7130696 US7016735 US7142909 US7103418 US7027852 US7035692 US7031769 US7149585 US7155295 US7072721 US7047084 US6983185 US7051738 US7010352 US7079900 US7104965 US7020531 US7047627 US6981314 US7151964 US7013170 US7013169 US6993392 US7072703 US7063708 US7076305 US7107100 US7120498 US7104988 US7016722 US7003354 US7029466 US7051419 US7125407 US7067327 US7149587 US7099712 US7123951 US7124493 US7184839 US7162310 US7212871 US7174221 US7283878 US7272448 US7231259 US7187983 US7302298 US7239922 US7289847 US7257449 US7313445 US7177697 US7212867 US7212855 US7280876 US7184842 US7191017 US7197362 US7308317 US7313444 US7242986 US7245973 US7302299 US7225035 US7200446 US7238883 US7286879 US7218971 US7181272 US7221981 US7225020 US7305270 US7212868 US7308318 US7181290 US7197361 US7239924 US7225021 US7308319 US7239923 US7174218 US7194315 US7257450 US7187975 US7187982 US7251532 US7187980 US7174222 US7218970 US7212869 US7164951 US7277762 US7236834 US7177701 US7158837 US7218972 US7248930 US7280875 US7242987 US7295881 US7242984 US7177702 US7187981 US7187984 US7225036 US7164952 US7225034 US7225025 US7254450 US7269461 US7248927 US7286884 US7191016 US7206642 US7277758 US7292889 US7184838 US7245967 US7158832 US7289846 US7277757 US7206641 US7231257 US7254451 US7286882 US7164948 US7184843 US7266413 US7299091 US7209787 US7191010 US7191015 US7191018 US7184837 US7177690 US7272449 US7263402 US7229469 US7236831 US7177680 US7251529 US7191012 US7158836 US7239912 US7231260 US7228181 US7239916 US7269457 US7203548 US7292890 US7206637 US7274966 US7239926 US7245972 US7221979 US7236822 US7263404 US7231252 US7167757 US7286883 US7177704 US7158839 US7242980 US7174219 US7305268 US7299096 US7216000 US7191008 US7263401 US7252090 US7167759 US7162309 US7194312 US7242977 US7187971 US7299092 US7274962 US7299097 US7228167 US7184829 US7190998 US7167750 US7302300 US7184840 US7158833 US7218968 US7234977 US7239925 US7203550 US7162308 US7257446 US7302294 US7211103 US7218966 US7209783 US7199110 US7175619 US7190993 US7310873 US7270669 US7294137 US7283856 US7167760 US7295875 US7276064 US7311704 US7272447 US7187970 US7182768 US7282213 US7181287 US7158838 US7225024 US7257434 US7229437 US7292895 US7292894 US7234225 US7248913 US7214197 US7204833 US7167743 US7306591 US7236819 US7207949 US7287995 US7255686 US7303526 US7187963 US7182764 US7194294 US7241283 US7392094 US7403823 US7353067 US7363091 US7406352 US7450993 US7353065 US7319905 US7463932 US7467017 US7447546 US7337005 US7337006 US7330765 US7383091 US7467016 US7433739 US7412289 US7347751 US7343200 US7369899 US7353066 US7363089 US7389149 US7321798 US7421300 US7324847 US7412290 US7349744 US7421299 US7463933 US7315763 US7386345 US7386350 US7389148 US7359755 US7330764 US7366574 US7343206 US7376468 US7317950 US7337008 US7392095 US7406350 US7437197 US7337009 US7369900 US7463934 US7349743 US7460907 US7321796 US7343202 US7369901 US7460906 US7389138 US7333857 US7349741 US7386351 US7450997 US7383090 US7444184 US7337004 US7324852 US7319906 US7395118 US7400926 US7444185 US7421295 US7450996 US7418298 US7429267 US7338522 US7346398 US7328072 US7444183 US7386341 US7450998 US7343204 US7386347 US7330760 US7330756 US7349739 US7398124 US7469162 US7463917 US7463924 US7315761 US7337012 US7454251 US7349742 US7337001 US7447545 US7319904 US7317951 US7451000 US7392093 US7409244 US7395113 US7398126 US7467012 US7460914 US7400931 US7458968 US7367992 US7463929 US7460913 US7337011 US7424319 US7366572 US7331960 US7384390 US7359756 US7379776 US7373207 US7418290 US7399300 US7442183 US7340309 US7330753 US7364546 US7462179 US7412284 US7546165 US7561922 US7596414 US7512447 US7532939 US7509172 US7477946 US7603178 US7515969 US7617006 US7551968 US7555352 US7539545 US7539546 US7499755 US7502651 US7617004 US7580758 US7542808 US7555349 US7617003 US7558632 US7509175 US7640064 US7555351 US7546166 US7623919 US7519435 US7515970 US7519432 US7499759 US7630772 US7571011 US7499756 US7526343 US7593777 US7610102 US7561924 US7587247 US7515968 US7529590 US7529591 US7551966 US7532938 US7483750 US7474924 US7519434 US7613524 US7499747 US7555350 US7628801 US7596409 US7610105 US7483753 US7529584 US7574263 US7546163 US7546164 US7486994 US7577481 US7483748 US7610106 US7532933 US7502650 US7529592 US7502652 US7489969 US7630769 US7512442 US7480532 US7634315 US7634319 US7512448 US7610104 US7565202 US7486995 US7620457 US7616991 US7499742 US7571001 US7529589 US7477947 US7522959 US7529582 US7623926 US7623925 US7590454 US7561920 US7489966 US7565198 US7499757 US7584004 US7515971 US7519433 US7617002 US7493173 US7499753 US7613515 US7587248 US7493175 US7499758 US7532937 US7571010 US7620454 US7536227 US7620458 US7620456 US7599736 US7536226 US7634317 US7571002 US7474923 US7617007 US7555347 US7548775 US7570997 US7496404 US7483754 US7610100 US7590455 US7637916 US7542807 US7547301 US7596415 US7473252 US7499749 US7483755 US7544197 US7590453 US7542803 US7477945 US7499750 US7519419 US7509174 US7590452 US7512446 US7559924 US7555343 US7608072 US7522950 US7565203 US7580756 US7604644 US7542802 US7489012 US7561907 US7570982 US7571012 US7555328 US7608064 US7497844 US7491181 US7725198 US7769472 US7702385 US7822482 US7840283 US7720550 US7643884 US7792590 US7801623 US7657326 US7844344 US7822486 US7706888 US7729783 US7797055 US7747322 US7856273 US7809446 US7751904 US7787961 US7840281 US7742810 US7840279 US7848823 US7835803 US7848822 US7729781 US7751905 US7680544 US7797059 US7657325 US7765015 US7725197 US7729780 US7783366 US7742828 US7672734 US7831311 US7848821 US7672735 US7657324 US7809442 US7822484 US7787962 US7826905 US7835804 US7797057 US7761170 US7844348 US7860576 US7860581 US7756588 US7787963 US7797058 US7676274 US7783365 US7706893 US7697995 US7647109 US7660635 US7676275 US7650186 US7676270 US7840261 US7792593 US7801625 US7831306 US7751897 US7672736 US7738970 US7818070 US7860572 US7711438 US7853333 US7797053 US7657323 US7831312 US7807925 US7769473 US7706894 US7818063 US7742818 US7725185 US7774071 US7697996 US7729771 US7792586 US7747335 US7747329 US7720540 US7693577 US7844343 US7715922 US7711429 US7702398 US7715923 US7756570 US7844347 US7738967 US7813812 US7848818 US7706886 US7697991 US7702399 US7657318 US7672730 US7783362 US7657322 US7801614 US7734343 US7684869 US7650190 US7672733 US7725196 US7831308 US7689290 US7715924 US7668602 US7711421 US7856260 US7738962 US7684866 US7751879 US7844338 US7778704 US7697984 US7801615 US7769462 US7840274 US7742811 US7769461 US7778703 US7778711 US7729759 US7840278 US7856274 US7756584 USR41463 US7805203 US7822488 US7801626 US7831305 US7835798 US7657315 US7853323 US7715916 US7856707 US7689260 US7783364 US7840282 US7822487 US7738953 US7842015 US7810233 US7715925 US7797029 US7774934 US7689292 US7809444 US7818039 US7653439 US7653438 US7751898 US7819871 US7715926 US7806836 US7828795 US7805188 US7734342 US7840269 US7668599 US7813796 US7725191 US7787954 US7655022 US7647097 US7669309 US7717848 US7792576 US7731653 US8041433 US7996090 US7949408 US7904163 US7899555 US7974707 US8055336 US7881783 US7894915 US8019442 US7865247 US7991481 US7890187 US8019440 US7979141 US7962220 US8019441 US7865249 US7912557 US7949411 US8060218 US8014875 US7890179 US7974705 US7917231 US7957819 US7996091 US8019419 US7873417 US7962224 US8032219 US8082042 US8078287 US7974710 US8010207 US7953499 US7883536 US7865246 US8005550 US8000810 US8005549 US7974704 US7908016 US8000784 US7937159 US7865248 US7917230 US8055355 US7979127 US7966076 US7904149 US7899552 US8060219 US8068920 US8014874 US8050773 US7917220 US8060217 US8027739 US8010208 US8027737 US7945337 US8014857 US8000805 US8036756 US7908017 US8027736 US7957806 US8019439 US7974700 US7962226 US7890188 US7933661 US7930039 US8050774 US7881806 US7899549 US7991478 US8010210 US8055354 US7890184 US8010200 US7991475 US7937160 US7894914 US7991480 US8024022 US7983768 US7881810 US8000802 US7970477 US8057486 US7890191 US7904179 US8019413 US8065020 US8000793 US7885715 US7953494 US7925358 US8050775 US8036752 US7912555 US7937156 US7894913 US8024050 US8014869 US8086317 US7974711 US8000798 US7983767 US7970479 US8019437 US7865250 US7904174 US7930037 US8052731 US7941228 US8024035 US8060207 US8086323 US7869882 US7962218 US8050768 US7974698 US8082040 US7894916 US7899532 US7873418 US8073546 US7945330 US8055357 US7949400 US8036751 US8036747 US7949395 US7899539 US8060206 US7890171 US7937144 US7917228 US7974675 US8036741 US7962221 US8046084 US7962225 US8065016 US7949393 US8046085 US8010204 US7986995 US8046075 US7953496 US7957810 US8019422 US8024039 US7974709 US7881809 US8005526 US7890189 US8043126 US8000796 US8014873 US7920921 US8086319 US7881796 US8065021 US7877147 US7908013 US8060211 US7974706 US7983756 US8032220 US7988507 US8019425 US7894911 US8032203 US7877150 US8024048 US7941202 US8065012 US7993352 US7974712 US7979105 US8078252 US7981119 US7970481 US8073547 US7930040 US7890192 US7904164 US7908010 US7957820 US8052676 US8032224 US7890186 US7976456 US7972367 US7979134 US8000807 US8007474 US8019421 US7904148 US8027740 US7865233 US7896873 US7887681 US7988690 US8075969 US7917187 US7935116 US8016767 US8180460 US8160714 US8224459 US8280507 US8116881 US8214058 US8103358 US8219213 US8224457 US8233994 US8321033 US8204605 US8160723 US8190267 US8112160 US8116875 US8332043 US8244376 US8321025 US8311646 US8175722 US8301260 US8271094 US8280515 US8099172 US8175719 US8244379 US8116880 US8295943 US8209031 US8112161 US8301269 US8214049 US8340785 US8170690 US8140170 US8155748 US8335572 US8260426 US8112154 US8103359 US8185213 US8239039 US8112157 US8103362 US8340783 US8244377 US8204606 US8103360 US8295945 US8145323 US8311645 US8321026 US8265761 US8229565 US8150536 US8290599 US8126571 US8135476 US8285396 US8195294 US8335571 US8190271 US8311644 US8326418 US8244375 US8229569 US8295933 US8271098 US8126568 US8260437 US8315704 US8295946 US8285394 US8214057 US8301267 US8332048 US8131359 US8099177 US8340782 US8224463 US8121705 US8271097 US8103350 US8224458 US8209033 US8209029 US8090450 US8306631 US8209037 US8190250 US8095225 US8326437 US8244346 US8140172 US8175724 US8170692 US8249721 US8249720 US8271096 US8150535 US8195307 US8195308 US8190269 US8271101 US8280526 US8121691 US8332046 US8311648 US8265765 US8095223 US8229574 US8165697 US8280514 US8244371 US8311649 US8260436 US8340784 US8108051 US8116883 US8224456 US8145325 US8204600 US8126569 US8306630 US8165687 US8112159 US8145326 US8285398 US8209035 US8233993 US8095224 US8285397 US8121684 US8155746 US8265773 US8249713 US8332047 US8301259 US8290595 US8239045 US8224453 US8204595 US8275464 US8180453 US8340780 US8332036 US8160702 US8324290 US8295947 US8219208 US8195300 US8175714 US8311635 US8195295 US8180461 US8301266 US8340760 US8116885 US8116872 US8285375 US8103361 US8340779 US8126560 US8185208 US8147486 US8233990 US8332035 US8126564 US8285395 US8150527 US8126562 US8170676 US8090448 US8121697 US8209032 US8239040 US8116876 US8219211 US8155747 US8315716 US8121695 US8126572 US8195266 US8131378 US8335568 US8150533 US8271100 US8285384 US8306626 US8229572 US8224417 US8090449 US8103357 US8280528 US8126570 US8337511 US8190253 US8195267 US8122596 US8105337 US8295902 US8280477 US8116868 US8131364 US8316537 US8340786 US8220467 US8221408 US8140162 US8136241 US8096990 US8181656 US8337431 US8204571 US8391994 US8577476 US8406895 US8538538 US8565896 US8543222 US8357187 US8509914 US8543202 US8457764 US8452416 US8406900 US8478430 US8554341 US8386054 US8594807 US8483842 US8620455 US8515558 US8504149 US8478428 US8494650 US8346374 US8374703 US8577453 US8380325 US8521307 US8457762 US8374705 US8565885 US8538548 US8577465 US8577466 US8560083 US8532780 US8548605 US8554342 US8504171 US8463392 US8442656 US8412340 US8494654 US8548602 US8437867 US8452419 US8489205 US8620425 US8386057 US8452418 US8494645 US8355783 US8615310 US8588940 US8406902 US8483846 US8615300 US8620452 US8417356 US8583254 US8588901 US8554337 US8442652 US8588938 US8442644 US8473073 US8463403 US8583257 US8412352 US8571682 US8498718 US8611999 US8509918 US8374697 US8442646 US8433424 US8423158 US8527067 US8583261 US8527066 US8571673 US8433412 US8423154 US8391995 US8612023 US8560087 US8543210 US8577468 US8577467 US8600505 US8457742 US8478427 US8437863 US8463400 US8396570 US8483830 US8406901 US8504170 US8577458 US8473074 US8515557 US8588941 US8571650 US8588939 US8412351 US8560086 US8364284 US8380319 US8433409 US8543220 US8577464 US8483845 US8521306 US8594761 US8532733 US8606370 US8417355 US8428750 US8504168 US8442647 US8369965 US8588921 US8355802 US8571665 US8473075 US8463404 US8577472 US8463399 US8554318 US8494649 US8406896 US8509915 US8606367 US8442657 US8359107 US8364274 US8364278 US8406897 US8478423 US8380324 US8560084 US8615308 US8442653 US8478408 US8538551 US8571685 US8401672 US8489202 US8442658 US8612021 US8374704 US8498721 US8473054 US8548604 US8396549 US8594811 US8396558 US8428747 US8548603 US8406886 US8620453 US8473076 US8359101 US8571657 US8369952 US8483828 US8364285 US8452421 US8538540 US8615293 US8412335 US8521294 US8467884 US8538533 US8620454 US8620457 US8423157 US8417345 US8463401 US8612005 US8538554 US8364280 US8425534 US8412348 US8430888 US8494655 US8538542 US8417334 US8600495 US8620423 US8551113 US8571676 US8509916 US8463353 US8433414 US8386056 US8606359 US8515560 US8612001 US8355788 US8386052 US8364271 US8583243 US8355800 US8364286 US8386006 US8433386 US8518092 US8460315 US8620459 US8386055 US8515549 US8543214 US8391996 US8401674 US8565851 US8571626 US8412302 US8586072 US8401635 US8548600 US8454529 US8359083 US8447416 US8594762 US8459268 US8620399 US8454593 US8380326 US8523823 US8534291 US8443810 US8355780 US8788061 US8630709 US8831750 US8805544 US8718791 US8812134 US8805543 US8666511 US8792999 US8644939 US8639354 US8718793 US8744598 US8655456 US8688233 US8676325 US8644942 US8676340 US8666504 US8897891 US8751018 US8781604 US8660661 US8712545 US8768484 US8731674 US8694126 US8812130 US8761899 US8781598 US8798773 US8812135 US8676331 US8670831 US8676347 US8914132 US8718784 US8644929 US8694128 US8718786 US8644956 US8805494 US8768475 US8655458 US8798770 US8694127 US8755906 US8644957 US8688236 US8751019 US8744595 US8751004 US8644955 US8700169 US8676351 US8632577 US8761903 US8700163 US8655459 US8694104 US8838255 US8676349 US8868213 US8700164 US8630718 US8666506 US8718795 US8744597 US8634934 US8660665 US8682444 US8781601 US8718777 US8781599 US8712546 US8774940 US8903508 US8788065 US8868214 US8738154 US8666507 US8639327 US8755905 US8849426 US8805534 US8805546 US8639344 US8761902 US8805547 US8652187 US8892217 US8630707 US8718788 US8676322 US8818517 US8676346 US8676342 US8792997 US8626314 US8700179 US8849414 US8862245 US8744596 US8666508 US8700183 US8676329 US8774913 US8700180 US8798767 US8634893 US8805541 US8676323 US8831748 US8805542 US8923985 US8903506 US8886335 US8649878 US8682450 US8706259 US8676341 US8892216 US8886336 US8812133 US8706243 US8644952 US8694123 US8666505 US8892214 US8868211 US8880193 US8880187 US8918177 US8818509 US8788055 US8886324 US8909354 US8831745 US8634921 US8897892 US8909355 US8914120 US8712547 US8918185 US8874239 US8903513 US8792996 US8874224 US8755909 US8774924 US8768476 US8781603 US8731669 US8874220 US8792994 US8914124 US8768465 US8649881 US8682430 US8666514 US8688231 US8812113 US8909352 US8744589 US8660662 US8918186 US8626301 US8868198 US8880176 US8874238 US8798766 US8818526 US8843202 US8849417 US8744584 US8923982 US8923988 US8862246 US8886338 US8660645 US8818527 US8918193 US8892211 US8670838 US8903512 US8849418 US8886323 US8831749 US8825177 US8744587 US8798758 US8849411 US8855776 US8918188 US8903509 US8903511 US8831730 US8849422 US8914121 US8792993 US8825155 US8923983 US8868191 US8761893 US8825181 US8897893 US8862225 US8909348 US8849410 US8918179 US8918172 US8892201 US8781591 US8914128 US8918173 US8630721 US8818504 US8774929 US8897890 US8897870 US8834545 US8792978 US8892208 US8918174 US8868208 US8892207 US8886340 US8886332 US8914130 US8923977 US8886339 US8676344 US8843216 US8923981 US8868210 US8886329 US8874232 US8909342 US8718789 US8838234 US8731660 US8781579 US8874233 US8874206 US8918187 US8712533 US8718782 US8874221 US8914125 US8788052 US8649870 US8718781 US8849406 US8886325 US8788066 US8666495 US8868207 US8923975 US8923974 US8660668 US8874236 US8843188 US8738135 US8788041 US8914092 US8864806 US8818515 US8712531 US8831742 US8914131 US8843211 US8909345 US8880188 US8897882 US8798753 US8825180 US8855777 US8825163 US8897875 US8874217 US8751005 US8903503 US8682447 US8914113 US8788058 US8855778 US8868209 US8897880 US8838256 US8903515 US8843213 US8843212 US8761900 US8788042 US8831737 US8688211 US8825179 US8694130 US8694124 US8721659 US8914116 US8886322 US8788043 US8831746 US8892215 US8886330 US8886305 US8894697 US8843208 US8712537 US8849413 US8788045 US8886327 US8868192 US8838248 US8862239 US8874222 US8886328 US8792988 US8886326 US8892209 US8880177 US8774934 US8862240 US8874234 US8862237 US8725271 US8909346 US8825160 US8755890 US8923989 US8868202 US8880189 US8909347 US8886303 US8660664 US8914118 US8849416 US8914117 US8897889 US8712544 US8691877 US8903507 US8788064 US8862242 US8712543 US8918175 US8843214 US8903502 US8897881 US8880190 US8914094 US8914093 US8918155 US8918156 US8918157 US8862233 US8868215 US8909337 US8818512 US8903495 US8855767 US8923969 US8788035 US8781605 US8781590 US8909353 US8903510 US8923970 US8874240 US8874235 US8812126 US8788040 US8849415 US8886276 US8886313 US8880186 US8649868 US8718792 US8788053 US8805545 US8849403 US8915926 US8903514 US8849424 US8894582 US8892200 US8855765 US8911451 US8774936 US8712553 US8788062 US8805538 US8886331 US8843200 US8923986 US8849419 US8903505 US8862243 US8880191 US8849395 US8750988 US8918987 US8849423 US8774939 US8849421 US8868206 US8862244 US8831739 US8692117 US8855789 US8897895 US8843196 US8880165 US8903493 US8653384 US8897853 US8818507 US8886337 US8905948 US8849369 US8849396 US8914096 US8918192 US8849425 US8914111 US8831740 US8920432 US8805527 US8781586 US8874204 US8725266 US8897888 US8923984 US8666471 US8880179 US8875391 US8887387 US8805467 US8744549 US8825130 US8831741 US8831744 US8874216 US8855768 US8744548 US8798769 US8814860 US8671566 US8805468 US8740846 US8903504 US8849420 US8909349 US8868173 US8825178 US8639312 US8825129 US8750957 US8874237 US8805466 US8768470 US8798772 US8910376 US8909329 US8894646 US8747351 US8855737 US8644903 US8886279 US8849392 US8909316 US8868212 US8923972 US8870773 US8758242 US8784714 US8692559 US8852287 US8733367 US8886277 US8876761 US8948884 US8934992 US8948883 US8954167 US9020606 US8929993 US8996133 US8972027 US8983622 US9072890 US8996114 US8948881 US8938310 US9089690 US9108043 US9216283 US9079018 US9220887 US9220891 US8954164 US9044592 US9014822 US8972025 US9037260 US9026229 US8942822 US9037265 US8948882 US9031671 US9014816 US8954166 US8983624 US8942829 US9002478 US8989872 US8929999 US9026231 US8938297 US8942816 US8938308 US9037235 US9002472 US8954158 US9037249 US8983625 US8965531 US9026228 US9026226 US8996119 US9042991 US8983626 US9037267 US9031664 US9037261 US9020604 US8983610 US8996116 US8972028 US9042998 US8965515 US8989862 US8983609 US8965516 US8954165 US9037248 US8958891 US8983608 US9008797 US8996111 US8934991 US8954168 US8958889 US8958881 US8989874 US8942805 US9042994 US9031656 US9011509 US8948873 US8954153 US9042988 US8934980 US8934977 US8989871 US9020611 US8942815 US9002459 US8938294 US9067071 US8996132 US9002468 US8929980 US8929997 US9002460 US8996118 US9042990 US8948872 US9031654 US9042987 US8965499 US9037262 US9042989 US9043000 US8996122 US8929992 US8948880 US9014812 US8983612 US9037244 US9037252 US8965522 US9026216 US8972015 US9008774 US8965529 US8958879 US8942821 US9031670 US9011508 US8958871 US9008800 US9031669 US8958878 US9026227 US8958890 US8942798 US9014813 US9020607 US8996128 US8983598 US8983623 US8965528 US9037256 US9008790 US9020612 US8934983 US9205252 US9042985 US9020610 US9008783 US9002462 US8977368 US9037231 US8942812 US9014815 US9037268 US9031665 US9162055 US8942820 US9031661 US8996123 US8989873 US9211408 US8996137 US8968376 US9002461 US9008796 US8942825 US8934975 US9008772 US8938300 US8977367 US9008782 US9037257 US8958880 US9037263 US8954152 US9026204 US9026205 US8934981 US9037243 US8948871 US8958893 US8948865 US9002477 US9031657 US8954157 US8948874 US8989861 US9031663 US9008799 US9020599 US9042983 US8977363 US8954144 US8996134 US8942810 US8996135 US8998914 US9020609 US8954162 US9002476 US8972023 US8934973 US9037251 US9031658 US9014820 US8989865 US9042999 US8954145 US8996136 US9002449 US8968377 US9220811 US9205255 US8983595 US9031653 US8965513 US8938309 US9114251 US9216290 US9114248 US9050462 US9020608 US9050456 US9155879 US9095698 US9220889 US9126044 US9044588 US9033869 US9084895 US9220885 US8929991 US9095703 US9089691 US9067058 US9089708 US9014800 US8942824 US9031662 US8977355 US8998929 US8934978 US9119957 US9192758 US9162056 US8954156 US9033996 US9008780 US9220890 US9020605 US8965535 US9056197 US9020601 US8942826 US8996100 US8934974 US9039594 US9205250 US8954163 US9017341 US8927876 US9002471 US8983627 US9037242 US9138580 US9056196 US8954149 US9180299 US8955211 US9079016 US8996126 US8934985 US9132268 US8996129 US9144673 US9002451 US9220899 US9031647 US9089692 US9037264 US8958892 US9126037 US9002463 US9089701 US9149629 US9050453 US9119543 US9089702 US9079019 US9119958 US9205259 US9205271 US9108042 US9108025 US9186502 US9126031 US9113270 US9132271 US9095709 US9192765 US9095713 US9216171 US9050455 US8989863 US9095722 US9192760 US9108057 US9078739 US9079035 US8974397 US8989870 US9095303 US9002474 US9199071 US8989859 US8944985 US9072913 US9144679 US9162051 US9205261 US9044155 US9155877 US9084665 US9072903 US9056206 US9211133 US9107636 US9011510 US9020574 US9037245 US9042980 US9031792 US9079038 US9211401 US9162060 US9220888 US9138577 US9179850 US9084883 US9008785 US8986382 US9107592 US8929990 US9089700 US8992517 US9162053 US9180295 US9149290 US9186499 US9162052 US8972026 US9044609 US8934956 US9042978 US9205272 US9084892 US9014808 US9022962 US9168368 US9061134 US8958862 US9044618 US8989868 US9132270 US8992523 US8954169 US9079037 US9220916 US9216298 US8942823 US9017325 US8926523 US9023038 US9101281 US9119968 US9180284 US8992409 US9084546 US9114009 US8948843 US9216286 US9205253 US9192409 US9174062 US9155881 US8951464 US8996130 US8938290 US8934965 US8925191 US9127991 US8989840 US9135400 US9061139 US9131900 US9179875 US9008747 US8966745 US9005189 US9037226 US8992522 US9099720 US8929988 US8942801

Magnetic
