# Supplementary material for: Technological Improvement Rates and Evolution of Energy-Based Therapeutics
Source: Front Med Technol. 2021 Sep 3;3:714140. doi: 10.3389/fmedt.2021.714140 (PMC8757806; doi:10.3389/fmedt.2021.714140)
Supplement: Supplementary file 3 [file Table_3.DOCX]

**Patent set for Magnetic energy-based therapeutics domain**

**(Granted between 1970-2015)**

US3658051 US3841306 US3841305 US3848588 US3890953 US3915151 US3921620 US3943912 US4056097 US4033054 US4105017 US4095588 US4066065 US4095587 US4177796 US4162672 US4233965 US4266533 US4266532 US4315503 US4330892 US4391270 US4428366 US4456001 US4454883 US4480596 US4489711 US4550714 US4510925 US4501265 US4556051 US4561426 US4548208 US4537181 US4527550 US4509219 US4616629 US4587957 US4587956 US4574809 US4693238 US4683873 US4674482 US4665898 US4641633 US4654574 US4723536 US4793325 US4757804 US4727857 US4765310 US4850340 US4889526 US4798194 US4838850 US4932951 US4940453 US4911686 US4974114 US4950221 US4921560 US5045050 US5066272 US5067940 US5030196 US4994015 US5061234 US5014699 US4994016 US4993413 US5047005 US4994014 US5058582 US5000178 US4989604 US5017185 US4983159 US5035017 US5010897 US5085626 US5170784 US5078674 US5106361 US5088976 US5160591 US5156587 US5131904 US5147284 US5123898 US5116304 US5100373 US5085627 US5084003 US5087336 US5099756 US5092835 US5137507 US5169380 US5139474 US5158526 US5135466 US5108359 US5161272 US5162037 US5183456 US5211622 US5195941 US5269747 US5181902 US5224922 US5267939 US5269746 US5192263 US5232433 US5269745 US5195940 US5267938 US5197940 US5226020 US5226185 US5314400 US5366435 US5330410 US5351389 US5318561 US5344384 US5314401 US5338286 US5304111 US5277692 US5368544 US5312321 US5295494 US5476438 US5415617 US5441495 US5401233 US5453073 US5437600 US5453072 US5387176 US5458558 US5453074 US5451199 US5450859 US5389061 US5429585 US5450858 US5518495 US5480373 US5527259 US5518496 US5556418 US5496258 US5480374 US5562597 US5538495 US5514072 US5529568 US5566685 US5544665 US5529569 US5669868 US5595564 US5697883 US5700234 US5665049 US5693004 US5689224 US5593379 US5642739 US5624373 US5658234 US5632720 US5667469 US5626099 US5743844 US5792040 US5752911 US5707334 US5842966 US5725471 US5766124 US5738625 US5733240 US5833600 US5718662 US5813970 US5769778 US5788624 US5707333 US5782743 US5738624 US5743843 US5803896 US5817000 US5813971 US5827170 US5807233 US5807232 US5830140 US5833594 US4549532 US5836018 US5779694 US5720046 US6007476 US5951459 US5984854 US6007477 US5997464 US5935054 US5857957 US5993375 US5877627 US6004257 US5882292 US5888185 US5871438 US5908444 US5967967 US5921244 US5965282 US6001055 US5984856 US5989178 US5950239 US6001071 US6024691 US6149577 US6132361 US6042531 US6162166 US6066084 US6123658 US6132362 US6099459 US6086525 US6048302 US6117066 US6083149 US6146324 US6032677 US6013021 US6155966 US6142927 US6119631 US6113530 US6129659 US6167313 US6015377 US6123657 US6050931 US6048303 US6053859 US6093143 US6126589 US6132360 US6135118 US6155967 US6139486 US6085355 US6266556 US6179770 US6186941 US6174276 US6179772 US6213933 US6261221 US6179769 US6280376 US6290638 US6210317 US6203486 US6309340 US6234953 US6312376 US6238333 US6200259 US6223750 US6179771 US6235251 US6328685 US6328684 US6217604 US6263878 US6231497 US6217504 US6245006 US6217505 US6267720 US6293900 US6306076 US6322491 US6258020 US6267719 US6224537 US6174277 US6231496 US6187031 US6332862 US6275996 US6198958 US6193577 US6461289 US6364824 US6425852 US6418345 US6468199 US6458071 US6402678 US6443883 US6434423 US6371905 US6500110 US6497648 US6450940 US6491620 US6443882 US6425851 US6447440 US6348033 US6344021 US6383129 US6447499 US6375324 US6416458 US6398713 US6488615 US6406418 US6394946 US6461288 US6398712 US6482147 US6440059 US6432036 US6379295 US6453204 US6406419 US6338347 US6461377 US6571123 US6503187 US6569078 US6641520 US6527697 US6527694 US6652443 US6572528 US6527696 US6558311 US6561968 US6527695 US6547713 US6551233 US6663556 US6537197 US6592509 US6524233 US6589159 US6520903 US6652444 US6558310 US6547194 US6592510 US6632168 US6579222 US6575893 US6635009 US6648812 US6626818 US6575892 US6663555 US6551234 US6652445 US6652446 US6663557 US6616595 US6612311 US6656108 US6547714 US6623419 US6544164 US6628979 US6626820 US6666813 US6611962 US6669623 US6786859 US6770022 US6733435 US6733434 US6733436 US6827681 US6741889 US6819210 US6699172 US6830544 US6692427 US6676591 US6780150 US6679827 US6761681 US6749596 US6712753 US6730014 US6679828 US6689044 US6776753 US6796937 US6679825 US6770023 US6702730 US6729336 US6707455 US6783504 US6902521 US6858000 US6899667 US6839595 US6849040 US6888486 US6939287 US6926659 US6926660 US6961620 US6853865 US6971984 US6971983 US6926661 US6955642 US6913663 US6960159 US6895630 US6930220 US7033312 US7008370 US7074175 US7155285 US7153256 US7087008 US7104947 US7097610 US7008369 US6997863 US7090636 US7081083 US7104946 US6991594 US7121996 US7150710 US7033329 US7282021 US7160240 US7276020 US7309309 US7258658 US7175587 US7258659 US7264585 US7294101 US7267644 US7160239 US7288062 US7186209 US7158835 US7179217 US7163505 US7297100 US7225812 US7174217 US7280861 US7189198 US7300452 US7218962 US7367935 US7407478 US7410469 US7338431 US7320664 US7367936 US7465269 US7422555 US7326170 US7399270 US7361136 US7370656 US7601116 US7494458 US7591776 US7614996 US7608035 US7601114 US7611453 US7588529 US7520848 US7553272 US7524276 US7566295 US7507198 US7625562 US7601115 US7603905 US7857747 US7771341 US7651459 US7819794 US7744524 US7819006 US7803104 US7824324 US7658704 US7744522 US7699768 US7785245 US7740574 US7744523 US7824323 US7713186 US7678041 US7727138 US7731648 US7711431 US7697981 US7951061 US7988613 US8047979 US8062204 US8021292 US8052591 US7963904 US7878965 US7918779 US8313421 US8267850 US8177702 US8265910 US8292796 US8118722 US8216121 US8303479 US8308627 US8147395 US8262556 US8137257 US8192346 US8246529 US8105228 US8303478 US8313422 US8308628 US8135472 US8251885 US8197465 US8608634 US8439816 US8388509 US8485960 US8517908 US8475354 US8523753 US8585570 US8469872 US8415123 US8460167 US8568287 US8465408 US8506468 US8500619 US8343026 US8435166 US8430805 US8602960 US8504159 US8496570 US8613695 US8506469 US8608633 US8585567 US8591392 US8585569 US8590537 US8594798 US8579786 US8827886 US8758216 US8808159 US8864641 US8845508 US8757166 US8771163 US8740764 US8882651 US8834341 US8777831 US8911342 US8868177 US8923535 US8838247 US8740765 US8657732 US8734316 US8840537 US8808158 US8795148 US8795147 US8647252 US8870737 US8827885 US8697181 US8805516 US8715150 US8894560 US8768455 US8840536 US8671935 US8691261 US9011310 US8961385 US9180305 US9033861 US9005103 US8968172 US8974365 US9067052 US9095694 US8932195 US8956274 US9020600 US8926490 US8932196 US8951183 US9180306 US9079010 US9005102 US9101751 US8968819 US8956273 US8961384 US9108038 US8944985 US8998791 US8979727 US9033860 US8936542 US9037247 US9005101 US9028391 US8968173 US9095696 US9174045 US8979726 US9020590 US9049987 US8977353 US9114054 US8931490 US9005100
