# Supplementary material for: Technological Improvement Rates and Evolution of Energy-Based Therapeutics
Source: Front Med Technol. 2021 Sep 3;3:714140. doi: 10.3389/fmedt.2021.714140 (PMC8757806; doi:10.3389/fmedt.2021.714140)
Supplement: Supplementary file 4 [file Table_4.DOCX]

**Patent set for Microwave/Radiofrequency energy-based therapeutics domain (Granted between 1970-2015)**

US3527227 US4228809 US4204549 US4271848 US4282887 US4311154 US4315510 US4375220 US4494539 US4534347 US4597379 US4641649 US4643186 US4776086 US4791930 US4785829 US4747416 US4744372 US4741348 US4817635 US4815479 US4800899 US4884580 US4798215 US4867175 US4841989 US4873995 US4860770 US4865047 US4860752 US4813429 US4891483 US4974587 US4945912 US4964415 US5025810 US5026959 US5057106 US5033478 US5027829 US5074859 US5073167 US5168880 USR33791 US5150717 US5143063 US5148814 US5234004 US5246438 US5295955 US5275597 US5370676 US5364392 US5344435 US5301687 US5300099 US5364336 US5358515 US5366490 US5370678 US5370675 US5354325 US5370644 US5344441 US5330518 US5370677 US5371342 US5385544 US5391196 US5413588 US5464437 US5464445 US5431648 US5470352 US5441532 US5404881 US5405346 US5507743 US5536267 US5531677 US5531676 US5509929 US5507791 US5514131 US5545137 US5501704 US5480417 US5569241 US5549639 US5558672 US5571154 US5503150 US5575811 US5492122 US5520684 US5529067 US5575788 US5542916 US5540655 US5540737 US5496271 US5531662 US5599345 US5599346 US5683384 US5672173 US5672174 US5660836 US5643335 US5603697 US5693082 US5620480 US5628771 US5683381 US5628770 US5649973 US5683382 US5782827 US5735847 US5728143 US5800484 US5810804 US5733316 US5728144 US5848986 US5800429 US5843077 US5823197 US5769846 US5755754 US5810803 US5800486 US5788692 US5769879 US5755663 US5843144 US5737384 US5800494 US5817049 US5728094 US5741249 US5827277 US5776176 US5755753 US5972026 US5863290 US5913855 US5928229 US5980517 US5951547 US5935123 US5925042 US5916241 US6002968 US5922013 US5944749 US5916240 US5865788 US5964791 US5861021 US5902251 US5987360 US5957969 US5899857 US5967976 US5919219 US5871524 US5974343 US5948011 US5931860 US5899932 US5964753 US6007570 US5992419 US5938692 US5904709 US6009351 US5957922 US5871525 US6083255 US6080150 US6071280 US6090105 US6132425 US6053937 US6059780 US6033401 US6097985 US6165174 US6132426 US6090106 US6067475 US6131577 US6123718 US6112110 US6104959 US6148236 US6156033 US6161049 US6134476 US6092528 US6050993 US6113595 US6122550 US6026331 US6071276 US6047216 US6119045 US6163726 US6051018 US6024743 US6077257 US6071279 US6063078 US6053913 US6102886 US6149647 US6123703 US6096037 US6106521 US6066135 US6066136 US6056744 US6033403 US6032078 US6109268 US6129726 US6122551 US6200333 US6283989 US6273907 US6299633 US6283988 US6306132 US6238389 US6216703 US6330478 US6292700 US6235023 US6238392 US6325796 US6210314 US6315778 US6231569 US6197021 USR37315 US6224593 US6175768 US6235022 US6241702 US6306133 US6312429 US6312428 US6241727 US6241753 US6223086 US6277116 US6334074 US6275738 US6283962 US6330479 US6319249 US6312427 US6325798 US6197023 US6258087 US6245066 US6208903 US6264654 US6254598 US6210405 US6190382 US6311090 US6190380 US6261311 US6319251 US6226553 US6289249 US6193714 US6315777 US6267758 US6245067 US6216041 US6181970 US6223085 US6188930 US6230060 US6245062 US6251109 US6233490 US6287302 US6287306 US6355033 US6352534 US6482203 US6471698 US6500175 US6447506 US6471699 US6402739 US6425912 US6364876 US6445957 US6453202 US6490488 US6425894 US6430446 US6500172 US6419653 US6470216 US6477426 US6447508 US6405090 US6377855 US6366818 US6436095 US6424869 US6440129 US6379349 US6464697 US6471696 US6383182 US6402743 US6428538 US6440130 US6402742 US6377854 US6458098 US6496737 US6419673 US6463331 US6500174 US6350276 US6471697 US6470219 US6496738 US6485486 US6428532 US6438424 US6387380 US6482202 US6440128 US6496736 US6451014 US6402744 US6427089 US6347251 US6413255 US6409723 US6497704 US6485489 US6497702 US6461378 US6381497 US6423058 US6381498 US6461353 US6408212 US6470217 US6562033 US6517536 US6587731 US6652520 US6582426 US6562031 US6645199 US6638277 US6604004 US6640120 US6533780 US6663624 US6632222 US6577903 US6522931 US6635055 US6551311 US6572639 US6660002 US6652516 US6558381 US6632221 US6641580 US6569159 US6660001 US6595989 US6514247 US6558378 US6508816 US6629971 US6572614 US6584361 US6645198 US6607529 US6605087 US6605085 US6592580 US6565561 US6562059 US6512956 US6587732 US6668197 US6540744 US6592579 US6517535 US6616655 US6666862 US6517568 US6623480 US6520185 US6640139 USR38299 US6503247 US6527768 US6544260 US6506189 US6562034 US6616657 US6629974 US6517537 US6547788 US6514250 US6575968 US6527769 US6611719 US6638275 USR38143 US6652519 US6620157 US6628990 US6663625 US6652518 US6503191 US6589238 US6682526 US6780182 US6689127 US6755827 US6807446 US6758856 US6689129 US6775575 US6673063 US6689131 US6832997 US6827716 US6740084 US6758847 US6692490 US6743226 US6723092 US6740082 US6673070 US6675050 US6788977 US6733496 US6751507 US6736835 US6673068 US6758846 US6685701 US6725095 US6780180 US6740108 US6723091 US6749605 US6780181 US6814732 US6690976 US6749607 US6699241 US6699243 US6702808 US6768925 US6749624 US6905498 US6974454 US6889694 US6899710 US6923806 US6932811 US6878147 US6979330 US6908464 US6918907 US6974455 US6958064 US6962588 US6969388 US6852110 US6958062 US6881213 US6958075 US6847848 US6846312 US6849073 US6932814 US6868290 US6855145 US6887237 US6979328 US6905496 US6974456 US6955675 US6872205 US6887238 US6869431 US6955173 US6972014 US6957108 US6960205 US6881214 US6976986 US6866663 US6866624 US6855144 US6878155 US6939344 US6923807 US6923805 US6962586 US6872206 US6893436 US6974450 US7074217 US7108696 US6994706 US7147632 US7128739 US7147633 US7041098 US7089064 US7150744 US7093601 US7004942 US7004938 US7022121 US7066935 US7041097 US7115126 US7090672 US7125407 US7033352 US7008420 US7069087 US7097641 US7052491 US7130697 US7004941 US7044949 US7118590 US7113832 US7008421 US7101369 US7099717 US6997925 US7094215 US7025767 US7020528 US7104989 US7118568 US7048733 US7006874 US7094232 US7043307 US7008419 US7160296 US7278991 US7311703 US7160292 US7197363 US7229438 US7163533 US7156841 US7223264 US7306595 US7189230 US7297143 US7261710 US7282050 US7226446 US7192427 US7292893 US7261712 US7220261 US7244255 US7300436 US7195629 US7163537 US7282061 US7238182 US7238183 US7165552 US7160294 US7306594 US7195626 US7179257 US7261709 US7311704 US7192429 US7241295 US7229436 US7267675 US7244254 US7301131 US7422586 US7419487 US7344533 US7318824 US7438714 US7396355 US7400930 US7326235 US7387627 US7318822 US7416551 US7374562 US7470271 US7465300 US7371234 US7377917 US7331960 US7354437 US7335197 USR40472 US7422583 US7393352 US7400929 US7387626 US7341586 US7452358 US7435250 US7468062 USR40559 US7416552 US7442192 US7338487 US7465301 US7625371 US7527623 US7594313 US7524318 US7604633 US7594913 US7585296 US7473251 US7507234 US7507238 US7588568 US7611508 US7497858 US7512445 US7632268 US7594915 US7517347 US7510555 US7513896 US7615049 US7585297 US7530979 US7507239 US7582050 US7494488 US7765010 USR42016 US7799019 US7826904 US7824394 US7736360 US7744596 US7833220 US7647123 US7699843 US7819318 US7771372 US7713268 US7799025 US7769468 US7655006 US7699838 US7769469 US7680542 US7691103 US7761169 US7699841 US7857809 US7811313 US7744592 US7815637 US7655005 US7837720 US8043287 US7992572 US7998139 US7875024 US7862559 US8035570 US8059059 US8068921 US8012148 US7976536 US7871408 US7864129 US7951140 US7955262 US7933660 US8007496 US7955368 US8073550 US7955326 US7933659 US8083732 US7896872 US7993337 US8068919 US7896909 US7912553 USR42724 US8057466 US7875028 US8292881 US8118808 US8246615 US8328800 US8201563 US8241273 US8224455 US8282632 US8287527 US8216227 US8235981 US8246614 US8192423 US8313486 US8292880 US8167878 US8298231 US8133217 US8267932 US8267929 US8226697 US8221405 US8137342 US8197477 US8216232 US8221414 US8157796 US8216223 US8313483 US8298223 US8147486 US8216226 US8175679 US8211101 US8308722 US8285392 US8221413 US8221410 US8202272 US8251991 US8187266 US8221417 US8100895 US8235988 US8137346 US8187312 US8303583 US8246616 US8150532 US8197475 US8182476 US8152799 US8277444 US8216215 US8328802 US8409187 US8353901 US8545493 US8469953 US8463396 US8473077 US8353902 US8355803 US8491579 US8430871 US8486057 US8343145 US8512328 US8568401 US8506563 US8465479 US8353907 US8535307 US8454593 US8586897 US8540710 US8374702 US8361068 US8409188 US8491580 US8617153 US8394092 US8343149 US8562598 US8409186 US8348947 US8398625 US8568399 US8523853 US8512326 US8579891 US8597289 US8430874 US8475449 US8574227 US8515554 US8529562 US8459268 US8449535 US8486063 US8414579 US8551084 US8540708 US8512332 US8535302 US8548599 US8359104 US8512321 US8469951 US8465482 US8480668 US8372068 US8518037 US8597290 US8353908 US8579895 US8617158 US8500726 US8535308 US8500732 US8491581 US8545492 US8398627 US8548600 US8348936 US8603088 US8603086 US8585696 US8430872 US8617145 US8444635 US8544473 US8617152 US8367959 US8442645 US8396564 US8906007 US8628527 US8870860 US8652127 US8764744 US8832927 US8628523 US8882759 US8876814 US8690869 US8852180 US8747398 US8672933 US8894640 US8740893 US8777939 US8834460 US8728067 US8888766 US8634929 US8734439 US8808281 US8655454 US8795268 US8808282 US8690868 US8882754 US8663213 US8643561 US8628529 US8882758 US8728070 US8852179 US8788060 US8808283 US8852186 US8906019 US8906009 US8728066 US8790335 US8834409 US8728069 US8758334 US8900229 US8768485 US8795264 US8784413 US8672934 US8882757 US8747351 US8679107 US8652123 US8632538 US8790340 US8647339 US8647332 US8632533 US8911439 US8679112 US8845632 US8911435 US8632532 US8628525 US8753340 US8858546 US8876815 US8805480 US8874230 US8827992 US8900231 US8685016 US8623006 US8784416 US8849367 US8758342 US8777942 US8853600 US8795267 US8753342 US8870863 US8814856 US8744594 US8740900 US8672932 US8652129 US8882760 US8825176 US8876818 US8894639 US8656924 US8894646 US8821486 US8623005 US8882764 US8728075 US8915913 US8827990 US8882753 US8900224 US8652022 US8876809 US8777937 US8894636 US8728068 US8685017 US9192437 US8968288 US9011421 US9024237 US9028473 US9031668 US8968290 US9113931 US8932281 US9168178 US9119648 US9192436 US8965536 US8974449 US9028476 US8992413 US9028475 US9055957 US9198724 US9198723 US8945144 US9113930 US9113925 US9113927 US9023024 US9039692 US9028474 US8968289 US8945111 US8968291 US9095359 US9023026 US8974450 US9023025 US9113926 US9033970 US9039693 US8974452 US9161811 US9084619 US8958888 US8992513 US9119647 US9017319 US9192440 US9144455 US9192422 US9028479 US9005190 US9033973 US8932291 US9005191 US9011473 US9119623 US9023031 US9005229 US9028477 US9033972 US9037259 US8956348 US8998894 US9011430 US9028472 US8992519 US9119649 US9127989 US9121774 US8992514 US8939971 US9005100 US8945109 US8986296 US9155589 US9005189 US9131984 US8968287 US8974829 US8977365 US8958887 US8954161 US9199071 US8979845 US9011422 US8968299 US9041616 US8944071 US8939970 US9039701 US8968295 US9179968 US8939912 US9072532 US9168085 US9023028 US9023038 US9095358 US9113929 US9060761 US8992522 US9149328 US8974451 US9204925 US9050098 US9138231 US8968292 US9039698 US8945104 US9017325 US8934989 US9039626 US9028485 US8956352 US9192438 US8968234 US9119952 US9014814 US9155583 US9007070 US8998901 US9151680 US8939969 US9033971 US8939913 US9017324 US9079011 US9031667 US8932287 US8936594 US9060782 US9125668 US8932290 US8998895 US8926589 US8992523 US9028487 US9008793
