# Supplementary material for: Technological Improvement Rates and Evolution of Energy-Based Therapeutics
Source: Front Med Technol. 2021 Sep 3;3:714140. doi: 10.3389/fmedt.2021.714140 (PMC8757806; doi:10.3389/fmedt.2021.714140)
Supplement: Supplementary file 5 [file Table_5.DOCX]

**Patent set for Optical energy-based therapeutic domain**

**(Granted between 1970-2015)**

US3658068 US3867948 US3900034 US4232678 US4246905 US4553546 US4556057 US4561440 US4538613 US4628931 US4576173 US4623796 US4617926 US4622972 US4592361 US4633866 US4640283 US4651739 US4692924 US4672969 US4697590 US4653495 US4671285 US4733660 US4761047 US4762131 US4765337 US4790310 US4729621 US4804240 US4830460 US4849859 US4932934 US4966450 US4926861 US4976706 US4930505 US4950266 US4968314 US4953549 US4945908 US4930504 US4951663 US4976705 US4905690 US4907132 US4950268 US4918319 US4966144 US4917084 US4973330 US5046494 US5050597 US5029581 US4994059 US5053006 US5074862 US4998930 US5019074 US5032124 US5057099 US5057102 US5062431 US4988348 US5009655 US4994058 US5066291 US4986628 US5000752 US5053033 US5002051 US4995691 US5125922 US5167228 US5152761 US5163426 US5169396 US5140984 US5150704 US5161526 US5163934 US5125925 US5152759 US5084043 US5086770 US5133708 US5151096 US5137018 US5095901 US5146917 US5154707 US5193526 US5265598 US5176133 US5257991 US5188631 US5282797 US5336217 US5292345 US5346488 US5344418 US5334190 US5312395 US5282842 US5304207 US5354323 US5336248 US5304212 US5358503 US5360426 US5354324 US5363347 US5354293 US5344433 US5344434 US5454782 US5405369 US5441530 US5405368 US5464436 US5456700 US5417653 US5409483 US5447528 US5466248 US5441531 US5454794 US5409480 US5409482 US5425754 US5445146 US5447527 US5445608 US5474528 US5568503 US5505726 US5387211 US5519534 US5562719 US5500009 US5483958 US5527350 US5503637 US5514168 US5545192 US5527349 US5489279 US5511563 US5549660 US5643334 US5658323 US5601619 US5620478 US5683437 US5700243 US5693049 US5609591 US5645578 US5626631 US5591219 US5632767 US5634922 US5683436 US5702432 US5643173 US5836999 US5707401 US5755751 US5797868 US5707403 US5766233 US5792184 US5725565 US5843143 US5824024 US5851225 US5725523 US5807232 US5738680 US5709645 US5720772 US5814078 US5837000 US5782895 US5720894 US5800478 US5849026 US5782896 US5800479 US5755752 US5792214 US5814008 US5776174 US5769878 US5830211 US5766234 US5868731 US5983900 US5871521 US5879376 US5964749 US5925034 US5998597 US5957959 US6007569 US5951596 US5919217 US5944748 US5861020 US5860967 US5916211 US5913884 US5989283 US5961543 US5865832 US5871520 US5957960 US5891186 US5989245 US5871522 US5976175 US5997569 US5913883 US5876427 US5989246 US6063108 US6027495 US6059819 US6143019 US6030411 US6048359 US6096066 US6135117 US6024760 US6045575 US6017360 US6094595 US6165205 US6074410 US6152951 US6013096 US6120529 US6074411 US6033431 US6146410 US6129748 US6162242 US6157854 US6146409 US6086558 US6063071 US6063109 US6107466 US6162211 US6139568 US6156028 US6138046 US6099554 USR36634 US6164787 US6149672 US6039728 US6110195 US6042603 US6149671 US6053936 US6021347 US6071302 US6156029 US6143018 US6084242 US6214033 US6312451 US6187029 US6249698 US6302900 US6254625 US6290712 US6280438 US6238424 US6306160 US6251127 US6269818 US6187030 US6214034 US6267779 US6273904 US6267780 US6251100 US6171331 US6200310 US6241752 US6231593 US6283958 US6299632 US6210425 US6267721 US6235046 US6214035 US6315772 US6238426 US6261310 US6183500 US6254594 US6200309 US6315775 US6221095 US6287300 US6251102 US6325793 US6290713 US6327506 US6273883 US6273905 US6217605 US6328760 US6290714 US6283956 US6319273 US6238425 US6171332 US6319274 US6312450 US6283986 US6494900 US6411852 US6428531 US6471692 US6387089 US6450170 US6416531 US6409719 US6443974 US6419642 US6471716 US6485484 US6425873 US6395016 US6443977 US6358242 US6398777 US6379376 US6358272 US6488697 US6454789 US6493570 US6350275 US6497718 US6443946 US6497719 US6402681 US6454790 US6443945 US6366802 US6443978 US6383209 US6379377 US6500198 US6413267 US6461866 US6402774 US6488698 US6413268 US6443975 US6491618 US6491715 US6436127 US6494901 US6454791 US6494899 US6363279 US6409744 US6364874 US6383177 US6464715 US6440155 US6364872 US6383175 US6547779 US6554824 US6533803 US6508813 US6582454 US6645230 US6537302 US6629971 US6530919 US6599286 US6503268 US6514277 US6663659 US6558411 US6632002 US6524330 US6593706 US6537303 US6629989 US6623513 US6623511 US6641600 US6514278 US6530918 US6626932 US6618620 US6572637 US6623512 US6549809 US6602275 US6663658 US6663620 US6610052 US6622729 US6524329 US6537301 US6572609 US6585751 US6503269 US6596016 US6551346 US6527764 US6582421 US6554853 US6558372 US6602245 US6599287 US6530944 US6645231 US6607550 US6610081 US6592611 US6537304 US6641599 US6517532 US6666878 US6602274 US6666856 US6622049 US6660025 US6641578 US6537305 US6800086 US6808532 US6736832 US6790205 US6695870 US6805701 US6830580 US6695871 US6702837 US6811564 US6755849 US6676655 US6673063 US6764501 US6796994 US6695835 US6811562 US6673097 US6718128 US6736807 US6743249 US6673096 US6802854 US6808523 US6811565 US6755820 US6717164 US6761729 US6685730 US6755848 US6702838 US6802853 US6749623 US6811563 US6807876 US6709446 US6806481 US6783541 US6746473 US6821289 US6689124 US6690976 US6796978 US6702808 US6692486 US6685729 US6673095 USR38670 US6719778 US6881212 US6890346 US6921413 US6899723 US6866678 US6887260 US6872221 US6942657 US6872220 US6875225 US6918923 US6913616 US6942685 US6974224 US6896693 US6936043 US6979327 US6918922 US6875226 US6960201 US6860896 US6936064 US6837900 US6936044 US6887261 US6878144 US6974451 US6913615 US6976985 US6942655 US6955684 US7135034 US6981971 US7033350 US7087074 US7074233 US7131989 US7014639 US7048731 US6989023 US7001413 US7123636 US7153299 US7015636 US7018395 US7041121 US7037325 US7107996 US7144249 US7090670 US7033381 US7101384 US7044959 US7108690 US7033382 US6997923 US7100615 US6991644 US7147653 US7001414 US7101385 US7110825 US7016718 US7150710 US7135033 US7101365 US7094252 US7097639 US7108689 US7141028 US7077544 US6984229 US7060061 US7097655 US7118588 US7070611 US7066941 US7018397 US6984228 US7090691 US6986782 US7125416 US7051738 US7147654 US7081128 US7144248 US7018396 US7108712 US7077840 US7094210 US7118589 US7261730 US7250047 US7214223 US7252677 US7220254 US7177695 US7201767 US7190109 US7201766 US7156866 US7229435 US7201765 US7288108 US7297154 US7311723 US7181271 US7156865 US7285129 US7252678 US7210817 US7232456 US7311722 US7288107 US7217281 US7179278 US7208007 US7276058 US7241306 US7214222 US7229467 US7198633 US7267673 US7167741 US7306620 US7223270 US7198624 US7282060 US7198634 US7304201 US7264629 US7241291 US7288106 US7223281 US7204846 US7258695 US7309348 US7303578 US7201764 US7275931 US7217267 US7261729 US7255691 US7194316 US7226470 US7449026 US7422599 US7351252 US7470270 US7364574 US7329252 US7328708 US7359601 US7374569 US7396361 US7438719 US7331953 US7373254 US7344555 US7458983 US7331952 US7329274 US7409954 US7331983 US7427289 US7431719 US7465312 US7364583 US7351242 US7354448 US7326199 US7353829 US7413572 US7351253 US7465307 US7465313 US7435252 US7402167 US7331964 US7329273 US7422598 US7354432 US7396354 US7344529 US7367342 US7620289 US7575589 US7578604 US7628939 US7597708 US7572284 US7524328 US7476888 US7479503 US7559945 US7479137 US7534255 US7526344 US7491222 US7488341 US7503927 US7604630 US7517344 US7494502 US7544204 US7513906 US7494503 US7485137 US7511031 US7498029 US7559944 US7753943 US7846191 US7763059 US7758621 US7695504 US7744590 US7704272 US7824394 US7736382 US7704246 USH2242 US7763016 US7758570 US7722656 US7794453 US7771374 US7763058 US7850720 US7842075 US7819910 US7762964 US7815630 US7678140 US7819007 US7740651 US7654949 US7753524 US7833257 US7778712 US7767208 US7691100 US7730893 US7691134 US7686839 US7722655 US7730894 US7815668 US7824435 US7799247 US7809417 US7785358 US7703458 US7988715 US7993382 US8028706 US7955367 US7988688 US8025686 US8083784 US7977658 US8012189 US7883534 US7992570 US7912554 US8043348 US7935139 US7972137 US7914523 US7878203 US8057525 US7918229 US7883535 US7886749 US8043349 US7942915 US7942916 US8080047 US7921853 US7985219 US7939078 US7979121 US8048135 US8025687 US7922751 US8021404 US8083785 US8048136 US8034091 US7947067 US7892268 US8002768 US7931028 US7887533 US7955366 US7935107 US7918779 US8021405 US7985500 US7993381 US7891361 US8025661 US7896908 US7976571 US7955365 US7963985 US7951181 US7991258 US8241343 US8105369 US8287578 US8097029 US8328858 US8246668 US8252033 US8162928 US8109981 US8236037 US8092507 US8128672 US8097028 US8089057 US8320725 US8303636 US8257416 US8206326 US8292935 US8286643 US8257412 US8308784 US8226696 US8337538 US8216289 US8104481 US8202307 US8105321 US8267982 US8246667 US8286641 US8167921 US8101888 US8328857 US8317848 US8187278 US8167920 US8206426 US8303635 US8273046 US8157807 US8313518 US8333756 US8118851 US8182473 US8142486 US8328796 US8236036 US8317847 US8246666 US8277444 US8208759 US8088153 US8240312 US8523924 US8506612 US8475507 US8518093 US8579951 US8419781 US8465532 US8496696 US8568463 US8591561 US8388669 US8474463 US8448644 US8449586 US8372128 US8480719 US8545543 US8518094 US8409262 US8435275 US8551147 US8535361 US8518095 US8574276 US8430920 US8465531 US8540757 US8545021 US8551149 US8366755 US8444683 US8371694 US8449587 US8409263 US8556951 US8571648 US8348985 US8435274 US8480720 US8506613 US8551104 US8562660 US8534292 US8348986 US8518027 US8388668 US8398692 US8439045 US8343142 US8465533 US8551150 US8496695 US8372127 US8475506 US8425577 US8486124 US8439927 US8535360 US8480722 US8460351 US8535359 US8608786 US8365752 US8454670 US8439959 US8579952 US8545022 US8393330 US8371695 US8486123 US8496650 US8585745 US8556950 US8430919 US8562658 US8398240 US8550625 US8486126 US8574279 US8551148 US8608787 US8486056 US8523926 US8545020 US8453651 US8562661 US8486125 US8454669 US8470010 US8545542 US8562659 US8387621 US8562657 USR43901 US8574277 US8574174 US8355793 US8435273 US8845703 US8795344 US8758332 US8845705 US8900284 US8702773 US8652188 US8870857 US8771328 US8715327 US8747447 US8761874 US8734498 US8668727 US8834545 US8632528 US8840654 US8702772 US8870934 US8701675 US8790382 US8642093 US8894696 US8770203 US8721696 US8910638 US8740957 US8681936 US8888830 US8864806 US8834546 US8685005 US8882819 US8882743 US8728136 US8814923 US8852255 US8623063 US8920409 US8740958 US8632576 US8894560 US8721695 US8900282 US8876810 US8647372 US8906360 US8808342 US8747446 US8662670 US8845704 US8702769 US8761561 US8702770 US8870740 US8709056 US8915948 US8784461 US8852254 US8864805 US8647373 US8821559 US8728135 US8702771 US8813756 US8858607 US8652185 US8801254 US8690933 US8906893 US8657862 US8838228 US8651111 US8715328 US8652186 US8894635 US8651112 US8814922 US8774885 US8771326 US8792978 US8778003 US8894697 US8814921 US8790381 US8882752 US8771327 US8758418 US8834544 US8900283 US8906079 US8882753 US8702640 US8814924 US8888829 US8790383 US8623002 US8672987 US8664198 US8778002 US8685071 US8721698 US8784462 US8685072 US8746253 US8979912 US9211214 US8945105 US9044596 US8998802 US9180309 US8983596 US9023089 US9056198 US9044595 US9192778 US8956345 US8968376 US9011509 US8980332 US9192780 US8974442 US9023019 US9168388 US8929978 US8926959 US8979914 US9149646 US9110082 US9034023 US8956363 US8945197 US8945101 US9028536 US8968375 US9028469 US8998914 US9050116 US9162078 US8967883 US8961577 US8945195 US8936591 US9023021 US8932338 US8940034 US9017392 US9180307 US8936630 US8992589 US8996131 US9020590 US8961580 US8956396 US9023092 US9040131 US8929979 US9204931 US8961579 US9078680 US9220915 US9005262 US8985119 US8961040 US9037247 US8979913 US8926677 US9205278 US8940033 US9011508 US9044598 US9138596 US8951296 US9023090 US8936629 US8945196 US8936028 US9039746 US9101759 US8926678 US9138593 US9144690 US8997752 US8938295 US9023091 US8934965 US8986358 US9138595 US8961578 US8926676 US9017391 US9320914 US9504607 US9492681 US9504847 US9522287 US9265967 US9333370 US9252369 US9227082 US9345902 US9308392 US9373807 US9381115 US9259594 US9517356 US9393438 US9339485 US9474811 US9463333 US9433799 US9399144 US9314648 US9409037 US9289622 US9539438 US9553422 US9545524 US5146917 US5154707 US5193526 US5265598 US5176133 US5257991 US5188631 US5282797 US5336217 US5292345 US5346488 US5344418 US5334190 US5312395 US5282842 US5304207 US5354323 US5336248 US5304212 US5358503 US5360426 US5354324 US5363347 US5354293 US5344433 US5344434 US5454782 US5405369 US5441530 US5405368 US5464436 US5456700 US5417653 US5409483 US5447528 US5466248 US5441531 US5454794 US5409480 US5409482 US5425754 US5445146 US5447527 US5445608 US5474528 US5568503 US5505726 US5387211 US5519534 US5562719 US5500009 US5483958 US5527350 US5503637 US5514168 US5545192 US5527349 US5489279 US5511563 US5549660 US5643334 US5658323 US5601619 US5620478 US5683437 US5700243 US5693049 US5609591 US5645578 US5626631 US5591219 US5632767 US5634922 US5683436 US5702432 US5643173 US5836999 US5707401 US5755751 US5797868 US5707403 US5766233 US5792184 US5725565 US5843143 US5824024 US5851225 US5725523 US5807232 US5738680 US5709645 US5720772 US5814078 US5837000 US5782895 US5720894 US5800478 US5849026 US5782896 US5800479 US5755752 US5792214 US5814008 US5776174 US5769878 US5830211 US5766234 US5868731 US5983900 US5871521 US5879376 US5964749 US5925034 US5998597 US5957959 US6007569 US5951596 US5919217 US5944748 US5861020 US5860967 US5916211 US5913884 US5989283 US5961543 US5865832 US5871520 US5957960 US5891186 US5989245 US5871522 US5976175 US5997569 US5913883 US5876427 US5989246 US6063108 US6027495 US6059819 US6143019 US6030411 US6048359 US6096066 US6135117 US6024760 US6045575 US6017360 US6094595 US6165205 US6074410 US6152951 US6013096 US6120529 US6074411 US6033431 US6146410 US6129748 US6162242 US6157854 US6146409 US6086558 US6063071 US6063109 US6107466 US6162211 US6139568 US6156028 US6138046 US6099554 USR36634 US6164787 US6149672 US6039728 US6110195 US6042603 US6149671 US6053936 US6021347 US6071302 US6156029 US6143018 US6084242 US6214033 US6312451 US6187029 US6249698 US6302900 US6254625 US6290712 US6280438 US6238424 US6306160 US6251127 US6269818 US6187030 US6214034 US6267779 US6273904 US6267780 US6251100 US6171331 US6200310 US6241752 US6231593 US6283958 US6299632 US6210425 US6267721 US6235046 US6214035 US6315772 US6238426 US6261310 US6183500 US6254594 US6200309 US6315775 US6221095 US6287300 US6251102 US6325793 US6290713 US6327506 US6273883 US6273905 US6217605 US6328760 US6290714 US6283956 US6319273 US6238425 US6171332 US6319274 US6312450 US6283986 US6494900 US6411852 US6428531 US6471692 US6387089 US6450170 US6416531 US6409719 US6443974 US6419642 US6471716 US6485484 US6425873 US6395016 US6443977 US6358242 US6398777 US6379376 US6358272 US6488697 US6454789 US6493570 US6350275 US6497718 US6443946 US6497719 US6402681 US6454790 US6443945 US6366802 US6443978 US6383209 US6379377 US6500198 US6413267 US6461866 US6402774 US6488698 US6413268 US6443975 US6491618 US6491715 US6436127 US6494901 US6454791 US6494899 US6363279 US6409744 US6364874 US6383177 US6464715 US6440155 US6364872 US6383175 US6547779 US6554824 US6533803 US6508813 US6582454 US6645230 US6537302 US6629971 US6530919 US6599286 US6503268 US6514277 US6663659 US6558411 US6632002 US6524330 US6593706 US6537303 US6629989 US6623513 US6623511 US6641600 US6514278 US6530918 US6626932 US6618620 US6572637 US6623512 US6549809 US6602275 US6663658 US6663620 US6610052 US6622729 US6524329 US6537301 US6572609 US6585751 US6503269 US6596016 US6551346 US6527764 US6582421 US6554853 US6558372 US6602245 US6599287 US6530944 US6645231 US6607550 US6610081 US6592611 US6537304 US6641599 US6517532 US6666878 US6602274 US6666856 US6622049 US6660025 US6641578 US6537305 US6800086 US6808532 US6736832 US6790205 US6695870 US6805701 US6830580 US6695871 US6702837 US6811564 US6755849 US6676655 US6673063 US6764501 US6796994 US6695835 US6811562 US6673097 US6718128 US6736807 US6743249 US6673096 US6802854 US6808523 US6811565 US6755820 US6717164 US6761729 US6685730 US6755848 US6702838 US6802853 US6749623 US6811563 US6807876 US6709446 US6806481 US6783541 US6746473 US6821289 US6689124 US6690976 US6796978 US6702808 US6692486 US6685729 US6673095 USR38670 US6719778 US6881212 US6890346 US6921413 US6899723 US6866678 US6887260 US6872221 US6942657 US6872220 US6875225 US6918923 US6913616 US6942685 US6974224 US6896693 US6936043 US6979327 US6918922 US6875226 US6960201 US6860896 US6936064 US6837900 US6936044 US6887261 US6878144 US6974451 US6913615 US6976985 US6942655 US6955684 US7135034 US6981971 US7033350 US7087074 US7074233 US7131989 US7014639 US7048731 US6989023 US7001413 US7123636 US7153299 US7015636 US7018395 US7041121 US7037325 US7107996 US7144249 US7090670 US7033381 US7101384 US7044959 US7108690 US7033382 US6997923 US7100615 US6991644 US7147653 US7001414 US7101385 US7110825 US7016718 US7150710 US7135033 US7101365 US7094252 US7097639 US7108689 US7141028 US7077544 US6984229 US7060061 US7097655 US7118588 US7070611 US7066941 US7018397 US6984228 US7090691 US6986782 US7125416 US7051738 US7147654 US7081128 US7144248 US7018396 US7108712 US7077840 US7094210 US7118589 US7261730 US7250047 US7214223 US7252677 US7220254 US7177695 US7201767 US7190109 US7201766 US7156866 US7229435 US7201765 US7288108 US7297154 US7311723 US7181271 US7156865 US7285129 US7252678 US7210817 US7232456 US7311722 US7288107 US7217281 US7179278 US7208007 US7276058 US7241306 US7214222 US7229467 US7198633 US7267673 US7167741 US7306620 US7223270 US7198624 US7282060 US7198634 US7304201 US7264629 US7241291 US7288106 US7223281 US7204846 US7258695 US7309348 US7303578 US7201764 US7275931 US7217267 US7261729 US7255691 US7194316 US7226470 US7449026 US7422599 US7351252 US7470270 US7364574 US7329252 US7328708 US7359601 US7374569 US7396361 US7438719 US7331953 US7373254 US7344555 US7458983 US7331952 US7329274 US7409954 US7331983 US7427289 US7431719 US7465312 US7364583 US7351242 US7354448 US7326199 US7353829 US7413572 US7351253 US7465307 US7465313 US7435252 US7402167 US7331964 US7329273 US7422598 US7354432 US7396354 US7344529 US7367342 US7620289 US7575589 US7578604 US7628939 US7597708 US7572284 US7524328 US7476888 US7479503 US7559945 US7479137 US7534255 US7526344 US7491222 US7488341 US7503927 US7604630 US7517344 US7494502 US7544204 US7513906 US7494503 US7485137 US7511031 US7498029 US7559944 US7753943 US7846191 US7763059 US7758621 US7695504 US7744590 US7704272 US7824394 US7736382 US7704246 USH2242 US7763016 US7758570 US7722656 US7794453 US7771374 US7763058 US7850720 US7842075 US7819910 US7762964 US7815630 US7678140 US7819007 US7740651 US7654949 US7753524 US7833257 US7778712 US7767208 US7691100 US7730893 US7691134 US7686839 US7722655 US7730894 US7815668 US7824435 US7799247 US7809417 US7785358 US7703458 US7988715 US7993382 US8028706 US7955367 US7988688 US8025686 US8083784 US7977658 US8012189 US7883534 US7992570 US7912554 US8043348 US7935139 US7972137 US7914523 US7878203 US8057525 US7918229 US7883535 US7886749 US8043349 US7942915 US7942916 US8080047 US7921853 US7985219 US7939078 US7979121 US8048135 US8025687 US7922751 US8021404 US8083785 US8048136 US8034091 US7947067 US7892268 US8002768 US7931028 US7887533 US7955366 US7935107 US7918779 US8021405 US7985500 US7993381 US7891361 US8025661 US7896908 US7976571 US7955365 US7963985 US7951181 US7991258 US8241343 US8105369 US8287578 US8097029 US8328858 US8246668 US8252033 US8162928 US8109981 US8236037 US8092507 US8128672 US8097028 US8089057 US8320725 US8303636 US8257416 US8206326 US8292935 US8286643 US8257412 US8308784 US8226696 US8337538 US8216289 US8104481 US8202307 US8105321 US8267982 US8246667 US8286641 US8167921 US8101888 US8328857 US8317848 US8187278 US8167920 US8206426 US8303635 US8273046 US8157807 US8313518 US8333756 US8118851 US8182473 US8142486 US8328796 US8236036 US8317847 US8246666 US8277444 US8208759 US8088153 US8240312 US8523924 US8506612 US8475507 US8518093 US8579951 US8419781 US8465532 US8496696 US8568463 US8591561 US8388669 US8474463 US8448644 US8449586 US8372128 US8480719 US8545543 US8518094 US8409262 US8435275 US8551147 US8535361 US8518095 US8574276 US8430920 US8465531 US8540757 US8545021 US8551149 US8366755 US8444683 US8371694 US8449587 US8409263 US8556951 US8571648 US8348985 US8435274 US8480720 US8506613 US8551104 US8562660 US8534292 US8348986 US8518027 US8388668 US8398692 US8439045 US8343142 US8465533 US8551150 US8496695 US8372127 US8475506 US8425577 US8486124 US8439927 US8535360 US8480722 US8460351 US8535359 US8608786 US8365752 US8454670 US8439959 US8579952 US8545022 US8393330 US8371695 US8486123 US8496650 US8585745 US8556950 US8430919 US8562658 US8398240 US8550625 US8486126 US8574279 US8551148 US8608787 US8486056 US8523926 US8545020 US8453651 US8562661 US8486125 US8454669 US8470010 US8545542 US8562659 US8387621 US8562657 USR43901 US8574277 US8574174 US8355793 US8435273 US8845703 US8795344 US8758332 US8845705 US8900284 US8702773 US8652188 US8870857 US8771328 US8715327 US8747447 US8761874 US8734498 US8668727 US8834545 US8632528 US8840654 US8702772 US8870934 US8701675 US8790382 US8642093 US8894696 US8770203 US8721696 US8910638 US8740957 US8681936 US8888830 US8864806 US8834546 US8685005 US8882819 US8882743 US8728136 US8814923 US8852255 US8623063 US8920409 US8740958 US8632576 US8894560 US8721695 US8900282 US8876810 US8647372 US8906360 US8808342 US8747446 US8662670 US8845704 US8702769 US8761561 US8702770 US8870740 US8709056 US8915948 US8784461 US8852254 US8864805 US8647373 US8821559 US8728135 US8702771 US8813756 US8858607 US8652185 US8801254 US8690933 US8906893 US8657862 US8838228 US8651111 US8715328 US8652186 US8894635 US8651112 US8814922 US8774885 US8771326 US8792978 US8778003 US8894697 US8814921 US8790381 US8882752 US8771327 US8758418 US8834544 US8900283 US8906079 US8882753 US8702640 US8814924 US8888829 US8790383 US8623002 US8672987 US8664198 US8778002 US8685071 US8721698 US8784462 US8685072 US8746253 US8979912 US9211214 US8945105 US9044596 US8998802 US9180309 US8983596 US9023089 US9056198 US9044595 US9192778 US8956345 US8968376 US9011509 US8980332 US9192780 US8974442 US9023019 US9168388 US8929978 US8926959 US8979914 US9149646 US9110082 US9034023 US8956363 US8945197 US8945101 US9028536 US8968375 US9028469 US8998914 US9050116 US9162078 US8967883 US8961577 US8945195 US8936591 US9023021 US8932338 US8940034 US9017392 US9180307 US8936630 US8992589 US8996131 US9020590 US8961580 US8956396 US9023092 US9040131 US8929979 US9204931 US8961579 US9078680 US9220915 US9005262 US8985119 US8961040 US9037247 US8979913 US8926677 US9205278 US8940033 US9011508 US9044598 US9138596 US8951296 US9023090 US8936629 US8945196 US8936028 US9039746 US9101759 US8926678 US9138593 US9144690 US8997752 US8938295 US9023091 US8934965 US8986358 US9138595 US8961578 US8926676 US9017391 US9320914 US9504607 US9492681 US9504847 US9522287 US9265967 US9333370 US9252369 US9227082 US9345902 US9308392 US9373807 US9381115 US9259594 US9517356 US9393438 US9339485 US9474811 US9463333 US9433799 US9399144 US9314648 US9409037 US9289622 US9539438 US9553422 US9545524
